# Impact of IgG response to malaria-specific antigens and immunity against malaria in pre-school children in Ghana. A cluster randomized, placebo-controlled trial

**DOI:** 10.1371/journal.pone.0253544

**Published:** 2021-07-20

**Authors:** Samuel Kofi Tchum, Samuel Asamoah Sakyi, Bright Adu, Fareed Arthur, Felix Boakye Oppong, Francis Dzabeng, Benjamin Amoani, Thomas Gyan, Kwaku Poku-Asante

**Affiliations:** 1 Department of Biochemistry and Biotechnology, College of Sciences, Kwame Nkrumah University of Science and Technology, Kumasi, Ghana; 2 Kintampo Health Research Centre, Kintampo-North, Ghana; 3 Department of Molecular Medicine, School of Medicine and Dentistry, Kwame Nkrumah University of Science and Technology, Kumasi, Ghana; 4 Department of Immunology, College of Health Sciences, Noguchi Memorial Institute for Medical Research, University of Ghana, Legon, Accra, Ghana; 5 Department of Biomedical Sciences, School of Allied Sciences, University of Cape Coast, Cape Coast, Ghana; Instituto Rene Rachou, BRAZIL

## Abstract

**Background:**

Iron fortification and micronutrient initiatives, specifically, vitamin A, and zinc supplementation are the most cost-effective developmental strategies against malnutrition and health emergencies in pre-school children. Iron-deficiency among pre-school children have been documented, however, studies evaluating the impact of immunoglobulin G (IgG) isotype responses among iron-fortified pre-school children in malaria endemic communities has not been assessed. We evaluated the impact of iron fortification on the IgG responses to GLURP R0, GLURP R2 and MSP3 FVO malaria-specific antigens among pre-school children in malaria endemic areas.

**Methods:**

This community-based, placebo*-*controlled, double-blinded, cluster-randomized trial study was conducted in Wenchi Municipal and Tain District of Bono Region. The trial was registered at ClinicalTrials.gov-registered trial (Identifier: NCT01001871). Ethical approval was obtained and informed consent were sought from each participant parents/guardian. For the current objective, 871 children aged 6–35 months were screened, from which 435 children received semi-liquid home-made meals mixed with 12.5 mg of iron daily (intervention group), and 436 received micronutrient powder without iron (placebo group) for 5 months. Standardized clinical and epidemiological questionnaires were administered and blood samples taken to measure IgG responses to GLURP R0, GLURP R2 and MSP3 FVO recombinant antigens using the Afro Immunoassay (AIA) protocol.

**Results:**

Baseline anthropometry, malaria diagnosis, anaemia and iron status, demographic features and dietary intake were identical among the groups (*p* > 0.05). After the intervention, there was no significant difference in the IgG response against GLUP R0, GLUP R2 and MSP3 FVO between the iron-containing micronutrient and placebo groups (*p* > 0.05). The iron-containing micronutrient powder group who were iron-sufficient or iron replete had significantly higher IgG response to GLURP R0 and GLURP R2 compared to iron-deficient and iron-deficiency anaemia in the same group (*p* < 0.05). The IgG responses to all the three malaria specific antigens were low among children without malaria episode but high among those with two and four episodes due to exposure differences.

**Conclusion:**

Iron fortification did not influence antibody response against endogenous malaria specific antigens among pre-school children in malaria endemic areas, however, IgG response to malaria specific antigens were high among children with sufficient iron status.

## Introduction

Malaria is a major cause of morbidity and mortality among pre-school children and a main contributor to anaemia [1–3]. In 2016, malaria accounted for over 216 million cases with 0.5 million deaths, of which 91% were from Sub-Saharan Africa [3]. About half of the malaria mortalities occurred among African pre-school children, with almost the same population being iron deficient [4].

Parasitized erythrocytes have numerous antigenic surface proteins that illicit antibody response [5–7]. Asexual malaria blood stage Merozoite Surface Protein (MSP) and Glutamate Rich Protein (GLURP) antigens elicit immunological response that prevent *in vitro* parasitaemia against malaria have gone through phase 1 vaccine trials and their efficacies are being tested in both phase 2 and 3 vaccine trials among pre-school children in Sub-Saharan Africa [6, 8–11].

Recombinant malaria antigens GLURP R0 protein containing the conserved non-repeat N-terminal region, (amino acids 25–514), GLURP R2 (amino acids 705–1178) of the carboxyl-terminal repeat region all expressed in *Escherichia coli* and the recombinant MSP-3 of the FVO strain expressed in *E*. *coli* [12–14]. GLURP R0, GLURP R2 and MSP3 FVO antigens are blood stage malaria vaccine candidates [15–17].

Recent iron supplementation strategies to prevent malaria and anaemia have only been partially effective and antibody responses to malaria specified antigens have been inconclusive, yielding conflicting results from malaria endemic and non-endemic areas [18, 19]. The effect of iron supplementation on the immunity of pre-school children remains contentious. Some studies observed that children on iron supplements are more protected against risk of malaria and anaemia whilst other studies concluded, it rather predisposes them to malaria infection [20].

Polymorphisms in the IgG_3_ hinge region has been known to influence antigens specific to IgG_3_ variants that mitigate the response to malaria and indirectly prevent anaemia [13]. Immunological assessment among iron-deficient pre-school children have been studied by various investigators, however, studies ascertaining the effect of immunoglobulin G (IgG) isotype responses among iron-fortified pre-school children in malaria endemic communities have not been reported, particularly regarding immunity of malaria antigen candidates, iron fortification and deficiency. Specific outcomes on the acquisition of natural protection during iron intervention at infancy and early childhood is not well understood.

In addition, factors modulating immune responses against malaria vaccine candidates that may help provide possible explanations to the sub-optimal efficacies observed are not well understood. The current study assessed the impact of iron-containing micronutrient powder on malaria-specific isotype antigens IgG; GLURP R0, GLURP R2 and MSP3 FVO among pre-school children.

## Materials and methods

### Study design, site and population

**Fig 1:**
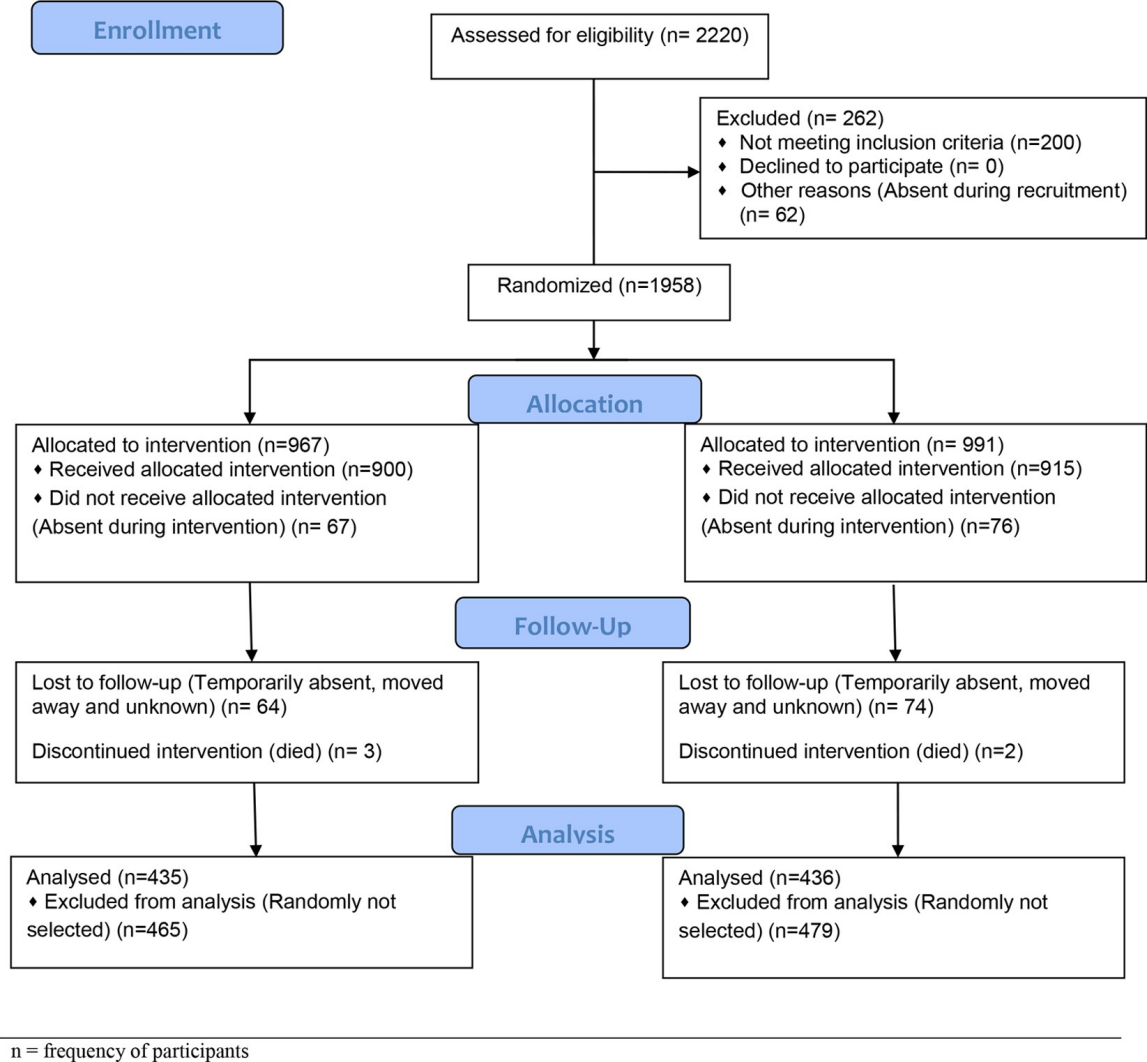
Flow diagram for participants’ selection and enrollment.

This community-based, placebo*-*controlled, double-blinded, cluster-randomized trial study was conducted in Wenchi Municipal and Tain District of Bono Region. Ethical approval was obtained and informed consent were sought from each participant parents/guardian. For the current objective, 871 children, aged 6–35 months were screened, from which 435 children received semi-liquid home-made meals mixed with 12.5 mg of iron daily (intervention group), and 436 received micronutrient powder without iron (placebo group) for 5 months. Standardized clinical and epidemiological questionnaires were administered and blood samples taken to measure IgG responses to GLURP R0, GLURP R2 and MSP3 FVO recombinant antigens using the Afro Immunoassay (AIA) protocol. Children with extreme anaemia (haemoglobin 70.0 g / L), severe malnutrition (weight-for-length z-score -3.0), on iron supplements during the past 6 months or had any chronic disease were excluded (Fig 1).

### Ethics approval, consent and recruitment of participates

Ethical approval was obtained from the Kintampo Health Research Centre Institutional Ethics Committee (KHRCIEC), (KHRC/IEC/FEA/2009-2), Ghana Health Service Ethics Committee. In addition, the Food and Drug Authority of Ghana and Hospital for Sick Children Research Ethics Board (REB), Toronto, Canada (REB File No.: 1000013476) also approved the study. The trial was registered at ClinicalTrials.gov-registered trial (https://clinicaltrials.gov/ct2/show/record/NCT01001871) [21]. The study was explained in local dialect to parents and guardians and informed consent was obtained from respective guardians before the children were enrolled as previously described [21, 22]. Qualifying children aged 6 to 35 months were enrolled and randomized (1:1) to receive either iron-MNP or without iron (MNP-Sprinkles® Mumbai, India). A cluster consisted of one or more households in the same compound with at least one child enrolled, and a cluster randomization design was used to prevent cross contamination between the groups through food sharing, using (in-house written programme) a computer-generated model [22]. The detailed comprehensive randomization and recruitment process has previously been reported [22].

### Specimen collection, processing and analysis

About 500 μl of finger or heel prick blood was collected from each participants into an ethylene diamine tetra-acetic acid (EDTA) tube at baseline (BL) and endline (EL) for haematological, malaria microscopy, acute protein process, and iron biomarker analyses as previously described [21, 22]. Briefly, at the start of the study, a malaria rapid diagnostic test (RDT) (Paracheck Pf 1 Device, Orchid Biomedical Systems, Verna, Goa, India) was used to screen the children; those who tested positive were treated for malaria, after which they were enrolled. Fifty microliters (50 μL) of the blood was used to prepare thick and thin blood smears for malaria parasitaemia and speciation. Methanol was used to fix thin blood films and the smears were stained with Giemsa for malaria microscopy investigation. Two separate microscopists read each sample slide and if the disagreement was greater than 50% between the two readers, a third microscopist was consulted. The haematology auto-analyzer (Horiba ABX Micros 60-OT-CT-OS-CS, Montpellier, France) was used to determine the full blood count (FBC) and the QuikRead 101 analyzer was used to determine C-reactive protein (CRP) (Orion Diagnostica, Espoo, Finland). A haematofluorometer was used to measure the zinc protoporphyrin (ZPP) in red blood cells (Model 206D, Aviv Biomedical Inc. Lakewood, NJ, USA). The remaining blood sample was centrifuged to obtained plasma and the following procedures used an indirect enzyme-linked immunosorbent assay (ELISA) to measure ferritin (Fn) (Spectro Ferritin S-22, Ramco Laboratories Inc. USA) and transferrin receptor (TfR) (TFC-94, Ramco Laboratories Inc. USA) levels as previously reported in details elsewhere [21, 22].

The IgG responses to GLURP R0, GLURP R2 and MSP3 FVO recombinant antigens was determined using indirect enzyme-linked-immunosorbent assays (ELISA) with a little modification made in the concentrations of the coating antigens and secondary / detection antibodies as previously described [7, 23]. Briefly, 100 μL of antigens was coated directly to each well in a 96-well microtiter ELISA plate (Maxisorp Nunc, Denmark) in coating buffer (plain PBS, pH 7.04) at 1.0 μg / mL for the recombinants and 5.0 μg / mL for the peptides. The coated plates were incubated overnight in a refrigerator at 2 to 8 ^0^C. Plates were then washed four times in washing buffer (PBS with 0.1% Tween-20 and 0.5 M NaCl) with 30 seconds incubation between each wash using the Biotek ELx 405 automated ELISA plate washer (Biotek Instruments, Winooski, VT; USA). The washed plates were padded dry on a tissue paper and blocked with 200 μL blocking buffer (PBS with 5% milk powder, 0.1% Tween-20) and incubated at room temperature in a humidified chamber for 1 hour. Plates were then washed four times in washing buffer and serum samples diluted at 1:200 in serum dilution buffer (PBS with 2.5% milk powder, 0.1% Tween-20 and 0.02% Na-azide) added at 100 μL / well in duplicates.

The plates with the samples were then incubated on a clinical rotator set at 200 rpm at room temperature for 2 hours in a humidified chamber after which they were washed four times in washing buffer and the appropriate secondary (detection) antibody for the specific antibody to be determined added at 100 μL / well. The plates with the conjugates were then incubated on a clinical rotator set at 200 rpm for 1 hour at room temperature in a humidified chamber after which they were washed four times in wash buffer and padded dry. The bound secondary antibody—(Peroxidase-labelled affinity purified antibody to human IgG (H+L)) was quantified by colouring with ready to use 100 μL / well of KPL ABTS Peroxidase Substrate System soln. A + soln. B in the ratio 1:1 and incubated in the dark for 30 minutes. Optical density (OD) was read at 405 nm with a reference at 620 nm in the microplate absorbance reader (DYNEX Technologies MRX Revelation Microplate Absorbance Reader, Inc. USA).

### Statistical analysis

The clinical and epidemiological data were entered into a Visual Fox Pro version 9.0 (Microsoft) data management programme and imported into STATA version 14.0 (Statcorp, Texas) and SigmaPlot version 11.0 for analysis. Study participants were grouped by their wealth indices into high or low socio-economic status. The distribution and comparison of proportions of the various total IgG isotype for each antigen were analyzed using the classical and nonparametric test of hypotheses. Test with *p*-values ≤ 0.05 were considered statistically significant. Kruskal-Wallis equality-of-populations rank test was used to determine whether total IgG isotype responses to the malaria antigens were associated to the risk of clinical malaria and anaemia in the cohort study.

## Results

### Demographic, clinical and socioeconomic characteristics of study participants

For the current objective, 871 children, aged 6–35 months were screened, from which 435 children received semi-liquid home-made meals mixed with 12.5 mg of iron daily (intervention group), and 436 received micronutrient powder without iron (placebo group) for 5 months. At baseline, there were no significant difference in the demographic and clinical characteristics between the groups (*p* > 0.05) except that the socio-economic status of household heads in the non-iron group was significantly higher than those in the iron group (*p* = 0.02)

The prevalence of low economic status of household heads was significantly higher in the iron group than in the non-iron group, whereas the prevalence of high economic status of household heads was significantly higher in the non-iron group (*p* = 0.02). (Table 1).

**Table 1:** Baseline characteristics among study participants.

Parameters	Iron Group (n = 435)	Non-iron Group (n = 436)	*p*-values
Total clusters number	388	386	
Size of cluster, median (range)	1(1–3)	1(1–3)	0.47
Age, mean [range], mo	19.6 [6–35]	19.4 [6–35]	0.74
Sex n (%)			0.97
Female	209 (48.8)	214 (49.1)	
Male	226 (51.2)	222 (50.9)	
Asymptomatic malaria parasitaemia prevalence, n (%)	431 (27.4%)	434 (28.3%)	0.75
Moderate anaemia Prevalence, n (%)	431 (42.7%)	434 (40.3%)	0.47
Parasitaemia, n, geometric mean, count/μL of blood	130, 2906.5	133, 2511.9	0.88
Household heads economic status, n (%)	413 (100)	423 (100)	0.02
High	116 (28.1)	152 (35.9)	
Low	297 (71.9)	271 (64.1)	

Frequency (n), percentages (%) of participants, age in months (mo), *p-*values are calculated by the Two-sample Wilcoxon rank-sum (Mann-Whitney) and 1-sided Fisher’s exact tests.

### Effects of iron fortification on malaria specific-antigens antibody responses

After the micronutrient powder (MNP) intervention, the IgG responses to the three recombinant malaria-specific antigens were similar between the iron-containing MNP and placebo groups (*p* > 0.05). However, the female participants in the iron group had significant higher MSP3-FVO antigens antibody responses than their male counterpart (*p* = 0.04) (Table 2).

**Table 2:** Effect of iron fortification on malaria antigen-specific IgG responses.

Geometric mean of IgG responses (Arbitrary units)					
Malaria Antigens	Iron group		Non-iron group		*p*-values
	n(GM)	95% CI	n(GM)	95% CI	
**GLURP R0**	408(741.6)	(627.7–876.1)	412(807.0)	(676.6–962.6)	0.29
Male	203(630.7)	(493.0–806.8)	212(683.9)	(526.1–889.0)	0.52
Female	205(871.3)	(695.9–1091.0)	200(961.7)	(761.4–1214.7)	0.38
***p*-values**	0.12		0.12		
**GLURP R2**	408(347.5)	(297.5–406.0)	412(361.2)	(305.5–427.0)	0.72
Male	203(319.0)	(257.1–395.7)	212(332.0)	(259.5–424.8)	0.78
Female	205(378.1)	(301.8–473.6)	200(395.0)	(314.9–495.5)	0.87
***p*-values**	0.18		0.26		
**MSP3 FVO**	408(750.5)	(447.8–869.4)	412(753.0)	(642.3–882.7)	0.73
Male	203(653.5)	(530.6–805.0)	212(693.2)	(693.2–877.6)	0.50
Female	205(860.6)	(699.2–1059.3)	200(851.4)	(690.5–1049.8)	0.81
***p*-values**	**0.04**		0.33		

n = Number of participants, 95% CI = 95% confidence interval, GM = geometric mean; Two-sample Wilcoxon rank-sum (Mann-Whitney) test for *p*-values.

### Effect of malaria-antigen specific IgG responses on anaemia and iron status

Iron-deficient was defined as lack iron reserves to meet functional or metabolic needs, iron-sufficient indicates excess iron reserves, iron-replete shows that enough iron reserves to meet functional or metabolic needs and finally, iron-deficiency anaemia is the final stage of iron deficiency characterized by a mean haemoglobin level below 10 g / L in preschool children [21, 22]. At baseline, there was no significant difference in the prevalence of iron deficiency between the children in the iron and non-iron arm (44.11% versus. 45.77%) [22]. The prevalence of endline iron deficiency was 24.5% (N = 443, 95% CI: 22.6% - 26.6%) [22]. Overall, endline prevalence of anaemia was 58.6% (N = 1059, 95% CI: 56.3% - 60.9%) and was more prevalent in children in the non-iron group than in those who received the iron intervention (62.63% versus. 55.52%, p = 0.003). Endline prevalence of moderate and severe anaemia were 52.7% (N = 951, 95% CI: 50.3%–55.0%) and 6.0% (N = 108, 95% CI: 5.0% - 7.2%) respectively [22]. Therefore iron fortification mitigated anaemia and improved iron status among the preschool children [22]. In the iron group, the iron-replete children had significantly higher GLURP R0 and R2 antigens IgG responses than the iron-deficiency anaemics (*p* < 0.05) (Table 3). The iron-sufficient children also had significantly higher GLURP R0 and R2 IgG responses compared to the iron-deficiency anaemics in the same group (*p* < 0.05). However, in the placebo group, the levels of GLURP RO, GLURP R2 and MSP3 FVO in either the iron replete children or iron deficiency anaemia were similar (Table 3).

**Table 3:** Malaria antigen-specific IgG responses on anaemia and iron status.

IgG Responses (Arbitrary unit)					
Malaria Antigens	n(Gm)	95% CI	n(Gm)	95% CI	*p*-values
	Iron-sufficient		Iron-deficient		
GLURP R0	630(852.3)	743.0–977.7	184(544.9)	420.5–706.0	0.001
GLURP R2	630(390.0)	344.0–442.1	184(251.5)	192.6–328.5	0.0003
MSP3	630(784.9)	695.3–886.0	184(635.3)	500.9–805.7	0.1
**Non-iron group**					
GLURP R0	300(875.4)	713.0–1074.8	110(625.4)	441.8–885.4	0.08
GLURP R2	300(389.5)	321.6–471.8	110(290.9)	205.6–411.7	0.06
MSP3	300(802.6)	670.4–960.7	110(649.1)	236.8–2571.7	0.29
**Iron group**					
GLURP R0	330(856.9)	277.9–3225.3	74(399.2)	104.4–1378.1	0.002
GLURP R2	330(356.7)	135.6–1067.5	74(125.9)	54.6–730.6	0.0005
MSP3	330(838.2)	347.2–2032.6	74(626.7)	272.1–1578.3	0.17
	**Iron-replete**		**Iron-deficient anaemia**		
GLURP R0	705(806.5)	707.1–919.8	109(574.5)	416.8–791.7	0.03
GLURP R2	705(373.5)	330.4–422.1	109(245.7)	177.9–339.3	0.01
MSP3	705(753.0)	670.3–845.9	109(718.1)	535.0–963.9	0.73
**Non-iron Group**					
GLURP R0	344(831.7)	684.7–1010.3	66(652.8)	426.2–999.9	0.19
GLURP R2	344(377.0)	313.1–453.8	66(283.9)	190.4–423.3	0.25
MSP3	344(773.5)	651.0–919.0	66(682.6)	454.4–1025.6	0.55
**Iron Group**					
GLURP R0	361(783.1)	655.1–936.1	43(472.1)	286.9–776.9	0.06
GLURP R2	361(370.2)	314.8–435.3	43(196.7)	112.8–343.1	0.01
MSP3	361(734.0)	626.8–859.6	43(776.1)	506.2–1189.9	0.9

n = Number of participants, 95% CI = 95% confidence interval, GM = geometric mean,. Two-sample Wilcoxon rank-sum (Mann-Whitney) and Kruskal-wallis rank tests for *p*-values.

### Effect of malaria-antigens specific IgG responses on malaria

**Fig 2:**
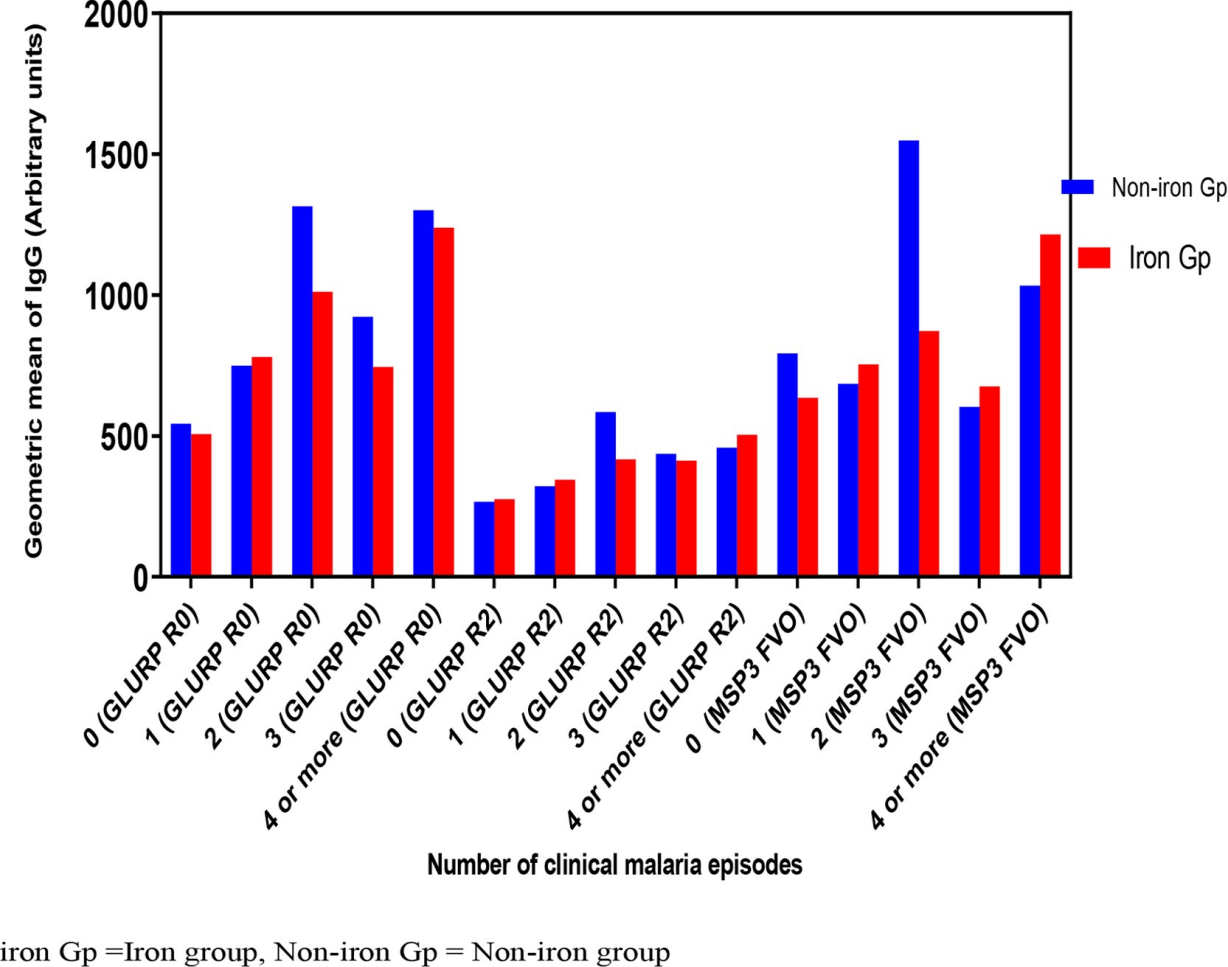
Antigen-specific IgG responses on the number of malaria episodes.

In iron group, the MSP3 FVO antigen IgG responses among the iron malarious children were significantly higher compared to their healthy counterparts (*p* = 0.05) (Table 4). However, in both groups, the malaria-specific antigens IgG responses to GLURP RO and GLURP R2 in either the malarious or healthy participants were similar (*p* > 0.05) (Table 4). In both the intervention and placebo groups, the IgG responses to GLURP R0 and GLURP R2 antigens were significantly higher in the malarious children relative to the healthy group (*p* < 0.05). In both groups, there was a significant rise in malaria specific-antigens IgG levels from episode zero to two (*p* < 0.05) but reduces at episode three and rose again from episodes four or more (Fig 2). When there was no malaria episode, IgG levels to all three antigens were the lowest, but they were the highest for episodes two and four (Fig 2).

**Table 4:** Antigen-specific IgG responses between malaria and healthy participant.

IgG Responses (Arbitrary units)					
Malaria Antigens	Malaria		Healthy		*p*-values
	n(GM)	95% CI	n(GM)	95% CI	
GLURP R0 (Non-iron)	299(937.0)	768.9–1142.0	113(543.5)	376.0–785.4	0.012
GLURP R0 (Iron)	294(858.6)	705.1–1045.6	113(506.6)	372.9–688.2	0.002
***p*-values**	0.49		0.48		
GLURP R2 (Non-iron)	297(405.7)	333.2–494.1	113(266.1)	194.2–364.7	0.023
GLURP R2 (Iron)	292(380.7)	318.0–455.9	114(275.2)	202.4–374.0	0.04
***p*-values**	0.57		0.71		
MSP3 FVO (Non-iron)	298(829.2)	688.7–998.3	113(620.7)	458.7–840.0	0.11
MSP3 FVO (Iron)	294(800.3)	666.7–961.0	114(635.8)	501.8–805.5	0.05
***p*-values**	0.83		0.82		

n = Number of participants, 95% CI = 95% confidence interval, GM = geometric mean; Two-sample Wilcoxon rank-sum (Mann-Whitney) test for *p*-values.

## Discussions

This study for the first time assessed the impact of iron fortification on IgG responses to GLURP R0, GLURP R2 and MSP3 FVO recombinant antigens among pre-school children in malarial endemic settings. The current study found that, iron fortification did not influence IgG response to GLUP R0, GLUP R2 and MSP3 FVO. Moreover, iron fortified children with good iron status (iron replete and iron sufficient) had higher IgG response against malaria specific antigens compared to those with poor iron status (iron deficient and iron deficiency anaemics).

Our findings is similar to studies by Priyanka and colleagues who conducted a randomized iron supplement controlled trial among Malawian children which compared prenatal and postnatal supplementation with multiple micronutrients, lipid-based nutrient supplementation, and observed that there iron supplementation has no effect on malaria antibody acquisition during infancy [24]. Likewise, other iron supplementation studies did not find significant improvement on the parameters of immunity between the intervention group and the controlled group [25–27].

Our study, further observed that the female participants were more protected from malaria due to differences in certain unknown exposures compared to their male counterparts irrespective of the study group but immunity was more significant among females with IgG responses against MSP3 FVO antigens compared to the males in the iron group. This observation was consistent with a study conducted in Malawi [28]. On the contrary, Kaddumukasa and colleagues reported that gender was not significantly associated with increase IgG response against GLURP, HRPII and MSP3 antigens in Uganda [29].

Research findings in recent past have indicated the important role iron plays in the immunological developments in children especially, during proliferation and maturation of immunized cells [30, 31].

In our study, the immunity against the malaria antigens waned as anaemia worsen among the children and even though appreciable IgG responses were observed in the iron group, the difference between the study groups were not significant.

The effect of anaemia severity on immunity clearly showed a major influence on humoral mediated immunity in our study, but knowledge on its mechanisms remain a mystery. Other studies have indicated that reduced levels of malaria-specific antibodies in iron-deficient children may indicate a decline in acquiring malaria immunity and/or a reduction in malaria exposure [32]. Though, the high burden of iron deficiency in African children is exacerbated by asymptomatic and febrile malaria [33]. Other findings, indicate that iron deficiency was related to immunity from mild clinical malaria among pre-school children [34]. Iron deficient and iron-deficient anaemic children in our study had lower immune response for MSP3 FVO antigen compared to the iron-sufficient and iron-replete study participants after receiving MNP with iron. Other findings suggested that humoral and cell-mediated immunogenic mechanisms were influenced by iron deficiency and iron deficiency anaemia resulting in their poor immunological response [27, 31, 35–37]. Possibly, the effects of Iron deficient and iron-deficient anaemia in children led to impairment on the maturation and proliferation of immune cells especially B and T-lymphocytes, thus increasing their vulnerability to other infections including malaria [31, 38].

We found that the iron-fortified children were more protected from malaria if they experienced two or more malaria episodes compared to children receiving MNP without iron. This findings corroborate with Tarun and Sachdev, who conducted systemic reviews of 28 randomized controlled trials involving 7892 children who were either receiving iron fortificants or placebo and concluded that there was no apparent detrimental effect on the general incidence of infection, especially malaria among the children, except a slight risk to diarrhea [20]. Similarly, Das and his colleagues also reported that iron supplementation improved immunity against malaria among children as their immunoglobulin responses increased in the iron group [27].

However, in our study, we observed that IgG response induced by the malaria antigens among healthy (non-malaria) children were lower compared to malaria parasitaemic participants in the presence of iron-fortified micronutrient powder. Thereby, suggesting that iron intervention may have played a significant role in controlling parasitaemia via inhibition of the growth or invasion of malaria parasites. Consequently, it is believed that IgG antibodies that prevent blood stage malaria parasites replication are mediated by naturally adapted and induced artificial immunity in children [39]. Furthermore, we found that children who had malaria in the iron-fortified group had higher immune response for MSP FVO than their healthy counterparts whilst immune response to MSP FVO in the placebo did not differ between the two. This finding was in accordance with a clinical trial by Mariama et al, in Burkina Faso, who found IgG immune response to MSP3 were associated with low incidence of malaria [40]. However, in both groups, our study found immune response to GLURP R0 and GLURP R2 to be similar between healthy children and those with malaria. This similarity may arise from previous exposures of the control group to malaria parasites prior to this study as these children live in malaria endemic villages.

Our study observed progressive protection from malaria as the number of clinical malaria episodes increased among the children in the iron group compared to children from non-iron group. Reasons for these findings maybe that, the mechanisms required to offer immunity against severe ailments are different from those that provide protection against mild diseases or infections with moderate episodes [41–43]. The main limitation of the current study was loss of children to follow-ups and inability to monitor coinfection during the follow-up.

## Conclusion

Iron fortification had no impact on IgG response to GLURP R0, GLURP R2 and MSP3 FVO malaria antigens, however, IgG response to malaria specific antigens were high among children with sufficient iron status.

## Supporting information

S1 Checklist Clinical trial check list. (DOC)

S1 File Research study plan. (PDF)

## List of abbreviations

GLURP R0: Glutamine Rich Protein R0
GLURP R2: Glutamine Rich Protein R2
MSP3 FVO: Merozoite Surface Protein (FVO)
MNP: Micronutrient Powder
IgG: Immunoglobulin G

## References

[1] Murphy SC, Breman JG. Gaps in the childhood malaria burden in Africa: cerebral malaria, neurological sequelae, anemia, respiratory distress, hypoglycemia, and complications of pregnancy. The American journal of tropical medicine and hygiene. 2001;64(1 suppl):57–67. 11425178 10.4269/ajtmh.2001.64.57

[2] Kyu HH, Pinho C, Wagner JA, Brown JC, Bertozzi-Villa A, Charlson FJ, et al. Global and national burden of diseases and injuries among children and adolescents between 1990 and 2013: findings from the global burden of disease 2013 study. JAMA pediatrics. 2016;170(3):267–87. doi: 10.1001/jamapediatrics.2015.4276 26810619 PMC5076765

[3] WHO. World Malaria Report 2017. World Health Organization (WHO). 2017.

[4] WHO. World Malaria Report 2015. World Health Organization (WHO). 2015.

[5] Faith H. A. Osier, G. F, Spencer D. Polley, Linda Murungi, Federica Verra, Kevin K. A. Tetteh, et al. Breadth and magnitude of antibody responses to multiple Plasmodium falciparum merozoite antigens are associated with protection from clinical malaria. INFECTION AND IMMUNITY. 2008;76, 2008:2240–8. doi: 10.1128/IAI.01585-07 18316390 PMC2346713

[6] Dodoo D, Aikins A, Kusi KA, Lamptey H, Remarque E, Milligan P, et al. Cohort study of the association of antibody levels to AMA1, MSP119, MSP3 and GLURP with protection from clinical malaria in Ghanaian children. Malaria journal. 2008;7(1):142. doi: 10.1186/1475-2875-7-142 18664257 PMC2529305

[7] David Courtin MO, Harm Huismans, Kwadwo Kusi, Jacqueline Milet, Cyril Badaut, Oumar Gaye, et al. Remarque, Robert Sauerwein, André Garcia, Adrian J. F. Luty. The quantity and quality of African children’s IgG responses to merozoite surface antigens reflect protection against Plasmodium falciparum malaria. PLoS ONE. 2009;4(10). doi: 10.1371/journal.pone.0007590 19859562 PMC2763201

[8] Dodoo D, Theisen M, Kurtzhals JA, Akanmori BD, Koram KA, Jepsen S, et al. Naturally acquired antibodies to the glutamate-rich protein are associated with protection against Plasmodium falciparum malaria. Journal of Infectious Diseases. 2000;181(3):1202–5. doi: 10.1086/315341 10720556

[9] Oeuvray C, Theisen M, Rogier C, Trape J-F, Jepsen S, Druilhe P. Cytophilic immunoglobulin responses to Plasmodium falciparum glutamate-rich protein are correlated with protection against clinical malaria in Dielmo, Senegal. Infection and immunity. 2000;68(5):2617–20. doi: 10.1128/IAI.68.5.2617-2620.2000 10768952 PMC97467

[10] Soe S, Theisen M, Roussilhon C, Druilhe P. Association between protection against clinical malaria and antibodies to merozoite surface antigens in an area of hyperendemicity in Myanmar: complementarity between responses to merozoite surface protein 3 and the 220-kilodalton glutamate-rich protein. Infection and immunity. 2004;72(1):247–52. doi: 10.1128/IAI.72.1.247-252.2004 14688102 PMC343946

[11] Cherif MK, Ouédraogo O, Sanou GS, Diarra A, Ouédraogo A, Tiono A, et al. Antibody responses to P. falciparum blood stage antigens and incidence of clinical malaria in children living in endemic area in Burkina Faso. BMC research notes. 2017;10(1):472. doi: 10.1186/s13104-017-2772-9 28886727 PMC5591548

[12] Adu B. Immunological and Genetic Correlates of Immunity to Plasmodium Falciparum Malaria: Department of Biochemistry and Biotechnology, Kwame Nkrumah University of Science and Technology, Kumasi; 2010.

[13] Opoku-Mensah J. Maternally transferred antibody levels and IgG3 hinge region length polymorphisms in the risk of clinical malaria in infants in a birth cohort at Kintampo, Ghana: University of Ghana; 2014.

[14] Theisen M, Vuust J, Gottschau A, Jepsen S, Høgh B. Antigenicity and immunogenicity of recombinant glutamate-rich protein of Plasmodium falciparum expressed in Escherichia coli. Clinical and diagnostic laboratory immunology. 1995;2(1):30–4. doi: 10.1128/cdli.2.1.30-34.1995 7719909 PMC170096

[15] Theisen M, Adu B, Mordmüller B, Singh S. The GMZ2 malaria vaccine: from concept to efficacy in humans. Expert review of vaccines. 2017;16(9):907–17. doi: 10.1080/14760584.2017.1355246 28699823

[16] Hamre KE, Ondigo BN, Hodges JS, Dutta S, Theisen M, Ayodo G, et al. Antibody correlates of protection from clinical Plasmodium falciparum malaria in an area of low and unstable malaria transmission. The American Journal of Tropical Medicine and Hygiene. 2020;103(6):2174–82. doi: 10.4269/ajtmh.18-0805 33124533 PMC7695051

[17] Adamou R, Dechavanne C, Sadissou I, d’Almeida T, Bouraima A, Sonon P, et al. Plasmodium falciparum merozoite surface antigen-specific cytophilic IgG and control of malaria infection in a Beninese birth cohort. Malaria journal. 2019;18(1):1–11. doi: 10.1186/s12936-018-2635-4 31185998 PMC6560827

[18] Tielsch JM, Khatry SK, Stoltzfus RJ, Katz J, LeClerq SC, Adhikari R, et al. Effect of routine prophylactic supplementation with iron and folic acid on preschool child mortality in southern Nepal: community-based, cluster-randomised, placebo-controlled trial. The lancet. 2006;367(9505):144–52. doi: 10.1016/S0140-6736(06)67963-4 16413878 PMC2367123

[19] Sazawal S, Black RE, Ramsan M, Chwaya HM, Stoltzfus RJ, Dutta A, et al. Effects of routine prophylactic supplementation with iron and folic acid on admission to hospital and mortality in preschool children in a high malaria transmission setting: community-based, randomised, placebo-controlled trial. The Lancet. 2006;367(9505):133–43. 10.1016/S0140-6736(06)67962-2 16413877

[20] Gera T, Sachdev H. Effect of iron supplementation on incidence of infectious illness in children: systematic review. Bmj. 2002;325(7373):1142. doi: 10.1136/bmj.325.7373.1142 12433763 PMC133452

[21] Zlotkin S, Newton S, Aimone AM, Azindow I, Amenga-Etego S, Tchum K, et al. Effect of iron fortification on malaria incidence in infants and young children in Ghana: a randomized trial. JAMA. 2013;310(9):938–47. doi: 10.1001/jama.2013.277129 24002280

[22] Tchum SK, Arthur FK, Adu B, Sakyi SA, Abubakar LA, Atibilla D, et al. Impact of iron fortification on anaemia and iron deficiency among pre-school children living in Rural Ghana. Plos one. 2021;16(2):e0246362. doi: 10.1371/journal.pone.0246362 33571267 PMC7877575

[23] Nebie I, Diarra A, Ouedraogo A, Soulama I, Bougouma EC, Tiono AB, et al. Humoral responses to Plasmodium falciparum blood-stage antigens and association with incidence of clinical malaria in children living in an area of seasonal malaria transmission in Burkina Faso, West Africa. Infect Immun. 2008;76(2):759–66. Epub 2007/12/12. doi: 10.1128/IAI.01147-07 ; PubMed Central PMCID: PMC2223475. 18070896 PMC2223475

[24] Barua P, Chandrasiri UP, Beeson JG, Dewey KG, Maleta K, Ashorn P, et al. Effect of nutrient supplementation on the acquisition of humoral immunity to Plasmodium falciparum in young Malawian children. Malaria journal. 2018;17(1):74. doi: 10.1186/s12936-018-2224-6 29415730 PMC5804088

[25] Thibault H, Galan P, Selz F, Preziosi P, Olivier C, Badoual J, et al. The immune response in iron-deficient young children: effect of iron supplementation on cell-mediated immunity. European journal of pediatrics. 1993;152(2):120–4. doi: 10.1007/BF02072487 8444218

[26] Sejas E, Kolsteren P, Hoeree T, Roberfroid D. Iron supplementation in previously anemic Bolivian children normalized hematologic parameters, but not immunologic parameters. Journal of tropical pediatrics. 2008;54(3):164–8. doi: 10.1093/tropej/fmm106 18211949

[27] Das I, Saha K, Mukhopadhyay D, Roy S, Raychaudhuri G, Chatterjee M, et al. Impact of iron deficiency anemia on cell-mediated and humoral immunity in children: A case control study. Journal of natural science, biology, and medicine. 2014;5(1):158. doi: 10.4103/0976-9668.127317 24678217 PMC3961924

[28] Barua P, Beeson JG, Maleta K, Ashorn P, Rogerson SJJMj. The impact of early life exposure to Plasmodium falciparum on the development of naturally acquired immunity to malaria in young Malawian children. 2019;18(1):1–12. 10.1186/s12936-019-2647-8 PMC6339377 30658632

[29] Kaddumukasa M, Lwanira C, Lugaajju A, Katabira E, Persson KE, Wahlgren M, et al. Parasite Specific Antibody Increase Induced by an Episode of Acute P. falciparum Uncomplicated Malaria. PloS one. 2015;10(4):e0124297. doi: 10.1371/journal.pone.0124297 25906165 PMC4408068

[30] Mascotti DP, Rup D, Thach RE. Regulation of iron metabolism: translational effects mediated by iron, heme, and cytokines. Annual review of nutrition. 1995;15(1):239–61. 10.1146/annurev.nu.15.070195.001323 8527220

[31] Hassan TH, Badr MA, Karam NA, Zkaria M, El Saadany HF, Rahman DMA, et al. Impact of iron deficiency anemia on the function of the immune system in children. Medicine. 2016;95(47). doi: 10.1097/MD.0000000000005395 27893677 PMC5134870

[32] Bundi CK, Nalwoga A, Lubyayi L, Muriuki JM, Mogire RM, Opi H, et al. Iron deficiency is associated with reduced levels of Plasmodium falciparum-specific antibodies in African children. Clinical Infectious Diseases. 2020. 10.1093/cid/ciaa728 PMC8246895 32507899

[33] Atkinson SH, Uyoga SM, Armitage AE, Khandwala S, Mugyenyi CK, Bejon P, et al. Malaria and age variably but critically control hepcidin throughout childhood in Kenya. EBioMedicine. 2015;2(10):1478–86. doi: 10.1016/j.ebiom.2015.08.016 26629542 PMC4634196

[34] Nyakeriga AM, Troye-Blomberg M, Dorfman JR, Alexander ND, Bäck R, Kortok M, et al. Iron deficiency and malaria among children living on the coast of Kenya. Journal of Infectious Diseases. 2004;190(3):439–44. doi: 10.1086/422331 15243915

[35] Rahmani S, Demmouche A. Iron deficiency anemia in children and alteration of the immune system. J Nutr Food Sci. 2014;4:333.

[36] Frost JN, Tan TK, Abbas M, Wideman SK, Bonadonna M, Stoffel NU, et al. Hepcidin-mediated hypoferremia disrupts immune responses to vaccination and infection. Med. 2021;2(2):164–79. e12. doi: 10.1016/j.medj.2020.10.004 33665641 PMC7895906

[37] Zimmermann MB. Global look at nutritional and functional iron deficiency in infancy. Hematology 2014, the American Society of Hematology Education Program Book. 2020;2020(1):471–7. 10.1182/hematology.2020000131 PMC7727574 33275751

[38] Ekiz C, Agaoglu L, Karakas Z, Gurel N, Yalcin I. The effect of iron deficiency anemia on the function of the immune system. The Hematology Journal. 2005;5(7):579–83. doi: 10.1038/sj.thj.6200574 15692603

[39] Pratt-Riccio LR, Bianco-Junior C, Totino PRR, Perce-Da-Silva DDS, Silva LA, Riccio EKP, et al. Antibodies against the Plasmodium falciparum glutamate-rich protein from naturally exposed individuals living in a Brazilian malaria-endemic area can inhibit in vitro parasite growth. Memórias do Instituto Oswaldo Cruz. 2011;106:34–43. doi: 10.1590/s0074-02762011000900005 21881755

[40] Cherif MK, Ouédraogo O, Sanou GS, Diarra A, Ouédraogo A, Tiono A, et al. Antibody responses to P. falciparum blood stage antigens and incidence of clinical malaria in children living in endemic area in Burkina Faso. 2017;10(1):472. 10.1186/s13104-017-2772-9 PMC5591548 28886727

[41] Marsh K, Kinyanjui S. Immune effector mechanisms in malaria. Parasite immunology. 2006;28(1‐2):51–60. doi: 10.1111/j.1365-3024.2006.00808.x 16438676

[42] Schofield L, Mueller I. Clinical immunity to malaria. Current molecular medicine. 2006;6(2):205–21. doi: 10.2174/156652406776055221 16515511

[43] Michon P, Cole-Tobian JL, Dabod E, Schoepflin S, Igu J, Susapu M, et al. The risk of malarial infections and disease in Papua New Guinean children. The American journal of tropical medicine and hygiene. 2007;76(6):997–1008. 17556601 PMC3740942

