# Supplementary material for: Impact of IgG response to malaria-specific antigens and immunity against malaria in pre-school children in Ghana. A cluster randomized, placebo-controlled trial
Source: PLoS One. 2021 Jul 20;16(7):e0253544. doi: 10.1371/journal.pone.0253544 (PMC8291688; doi:10.1371/journal.pone.0253544)
Supplement: S1 File — (PDF) [file pone.0253544.s002.pdf]

2. SPECIFIC AIMS

This RFA is intended to address "those factors affecting the safe and effective use of iron interventions for the prevention and treatment of iron deficiency and anemia in women of reproductive age (including adolescent girls), infants, and children, particularly in areas of endemic malaria". One of the three core priority areas described in the RFA is the category of "interventions". The RFA asks the question, "What are the safest and most effective interventions to prevent and treat iron deficiency in women, infants and children in areas of endemic malaria?" The current proposal specifically addresses the terms of the RFA by proposing the use of encapsulated ferrous fumarate, supplied in a powder form with other essential micronutrients, as an effective and potentially safer alternative to syrups or drops for providing iron to infants and young children living in regions where the burden of malaria is high.

In 2006, The World Health Organization (WHO) and United Nations Children's Fund (UNICEF) released a joint statement recommending that in malaria endemic areas, iron supplementation be targeted only to those children who are anemic and at risk of iron deficiency. As well, these children should receive concurrent protection from malaria and other infectious diseases through prevention and effective case management. The statement went on to state that "these conclusions should not be extrapolated to fortification or food-based approaches for delivering iron, where the patterns of iron absorption and metabolism may be substantially different. The Joint Statement did not provide recommendations regarding 'point-of-use' fortificants (i.e. single-dose powdered mineral and vitamin supplements to be sprinkled on to home-made foods at the table).

In reality, it is neither practical nor feasible to meet the population-wide screening requirements as outlined in the WHO/UNICEF Joint Statement (above), especially in under-developed countries. There simply are no screening tools for anemia and iron deficiency that can be practically used and, indeed, the cost of screening for hemoglobin alone is often higher than the cost of providing iron supplements. As a result, for the past 2-3 years, iron deficiency anemia has remained largely an unresolved nutritional problem in areas of endemic malaria. It is estimated, for example, that the prevalence of iron-deficiency anemia is as high as 60-65% in children under age 24 months in West Africa. In our past research, we have demonstrated in Ghana that the use of powdered minerals and vitamins, including microencapsulated iron will reduce anemia rates by up to 60%. However, because of the uncertainty around the use of iron in high malaria burden areas, the implementation (scaling-up) of these results is virtually at a stand-still.

Given that we have already demonstrated the efficacy of encapsulated iron (as ferrous fumarate) in lowering the burden of anemia, the primary objective of this research proposal is to determine the impact of providing encapsulated iron (as a powder added to complementary foods) on the susceptibility to clinical malaria among anemic and non-anemic infants and young children (6-24 months of age) living in a high malaria burden area. Due to the well documented tendency towards higher mosquito bite-rates in the rainy season, we will study two unique cohorts, one in the dry and one in the wet season. Certain secondary variables, such as iron and anemia status and breastfeeding rates may influence the primary outcome, thus we will concurrently collect data on these variables throughout the intervention.

Our secondary objectives are to determine the impact of this iron intervention on the *severity* of clinical malaria by documenting parasite counts and hospital admission rates, as well as differences in secondary complications of malaria infections, such as death, cerebral malaria, pneumonia, dehydration, and diarrhea. Although the current study is not powered to show causation between the intervention and the secondary outcomes, it is anticipated that the proposed research will help address the confusion that has been generated regarding the safe and appropriate use of iron supplements in high malaria transmission areas. If it is demonstrated that the provision of iron as a powder added to food does not have an adverse effect on malaria incidence and has a positive impact on anemia rates, then this type of iron delivery system can possibly be recommended for scale-up in Ghana and in other countries with a similar malaria burden. Conversely, if we

demonstrate adverse effects, especially in iron replete children, then further research is needed to identify inexpensive and non-invasive methods to screen children for anemia before iron is provided. **The long term goal of the proposed research is to develop new evidence to inform global policy and ultimately guide the implementation of programs to prevent and treat iron deficiency disorders in malaria endemic regions.** These goals coincide directly with the stated objectives of this RFA.

3. BACKGROUND AND SIGNIFICANCE

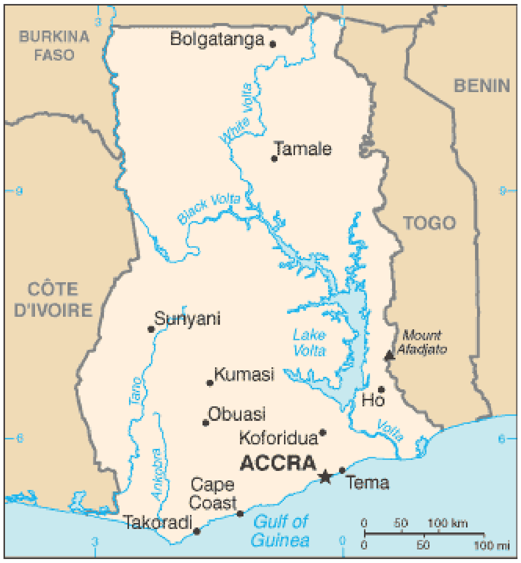

Ghana as the Proposed Study Site
Ghana, with an estimated population of 23 million, is located in Western Africa, bordering the Gulf of Guinea, between Cote d'Ivoire and Togo. Well endowed with natural resources, Ghana has roughly twice the per capita output of the poorest countries in West Africa. Even so, Ghana remains heavily dependent on international financial and technical assistance. Gold and cocoa production and individual remittances are major sources of foreign exchange. The domestic economy continues to revolve around agriculture, which accounts for about 35% of the gross domestic product and employs about 55% of the work force, which is mainly made up of small landholders.

Approximately 37.8% of the population is between 0-14 years of age (male 4,470,382/female 4,360,359). The birth rate is 29.22 births/1,000 population, while the death rate is 9.39 deaths/1,000 population (2008 est.). The total infant mortality rate is 52.31 deaths/1,000 live births (male: 56.64 deaths/1,000 live births; female: 47.85 deaths/1,000 live births [2008 est.]). Ghana is considered to be one of the most progressive countries in sub-Saharan Africa.

The value of performing this research in Ghana is three fold:

(i) There has been a well-established and successful research collaboration between the Government of Ghana (Kintampo Health Research Centre) and the Hospital for Sick Children, University of Toronto since 1998;

(ii) Malaria and anemia remain the most important causes of death and morbidity in Ghana; and

(iii) The vital capacity-building potential of joint projects, such as the one described in this application, goes well beyond the life of this project.

3.1 Background and Gaps

Global prevalence and impact of iron deficiency anemia

Iron deficiency and iron deficiency anemia (IDA) are the most prevalent micronutrient deficiencies on a worldwide basis, especially in developing countries. The impact of severe IDA can have mortal consequences, since without adequate hemoglobin, the brain and body become deprived of oxygen and, if allowed to continue, death may ensue. While the impact of mild and moderate IDA on child development and immune function remain areas of fertile research, there are currently no firm conclusions on either short- or long-term effects (1). There is agreement, however, that the prevention of iron deficiency and iron deficiency anemia (from mild to severe) are public health priorities, based on their potential negative impact on the health of children. There are experimental animal models that have examined potential mechanisms for the role of iron in brain development, and human cohort studies which have documented short- and long-term effects of mild-moderate IDA on impaired cognitive and motor development. Although the human cohort studies all suffer from an unavoidable design bias, since one cannot purposely assign (randomize) children to become anemic, those that have been conducted in different countries under different conditions have generally

shown similar adverse outcomes on school achievement and measures of cognition and learning. It has also been suggested that in terms of the impact of iron on the developing brain, the first two years of life are a ‘window of opportunity’, and once the window is closed the impact may not be reversible.

Despite the uncertainties around the impact of IDA on the health of children, governments and United Nations agencies continue to place high priority on the prevention and treatment of IDA. Even private ‘think-tanks’ like the Copenhagen Consensus have recognized the importance of controlling IDA. For example, in their most recent session, they ranked the control of micronutrient deficiencies as the number one global challenge, and placed iron fortification as the third most important solution due to their extremely high ratio of benefits to costs (2). And finally, the fourth Millennium Development Goal of “reducing the under-five child mortality to one third by 2015” is at least indirectly related to the control of IDA (3).

Global prevalence and impact of malaria

Nutritional intervention programs have generally demonstrated that the provision of iron supplements can enhance child development (4, 5) and reduce the prevalence of severe anemia; however, there is some evidence to suggest that iron supplementation (in the form of syrups, drops or pills often provided in a post-prandial state) results in high levels of malaria parasitemia (6, 7), increased rates of malaria, as well as pneumonia and diarrhea (7-10). In contrast, the most recent systematic review (2002) does not generally support an increased risk of malaria attack rate or severity associated with iron supplementation (pooled OR significant for malaria + smear = 1.43 [1.08-1.91], but not significant when adjusted for baseline malaria smear = 1.24 [0.98-1.57]) (10). Clearly multiple factors contribute to the complex etiology of anemia in high malaria burden areas, but iron status and malaria infection are the strongest predictors of hemoglobin concentration (11). Recent research has suggested that haptoglobin might also influence hemoglobin levels in an environment of malaria-induced hemolytic stress. Research by Atkinson and colleagues suggest that the $Hp^{2/2}$ genotype is a risk factor for childhood anemia in malaria-endemic countries. The authors found that average hemoglobin levels fell over the malaria season, and children who had the $Hp^{2/2}$ genotype had the greatest drop compared to other children (11). These findings suggest that a child’s haptoglobin type may be an important influence on whether that child gets anemia in areas where malaria is very common

The relationship between iron and malaria has important implications because malaria is a tropical parasitic disease that contributes significantly to morbidity and mortality rates in many parts of the world. Recent estimates from the 2005 World Malaria Report were around 350-500 million clinical disease episodes per year (12). A large proportion of the global malaria burden is concentrated in Africa, where approximately 60% of clinical cases and over 80% of malaria-related deaths occur. Further, most of the Africans who die from malaria each year are children under five years of age (13). Studies conducted in the north-western African country of Ghana have revealed that malaria can account for more than 44% of reported outpatient visits (12), and that the majority of these cases tend to occur during the wet season (June-October) (14, 15).

Impact of iron supplementation on malaria incidence and/or severity in Pemba, Zanzibar

A recent community-based randomized controlled trial, by Sazawal et al, demonstrated increased morbidity and mortality in infants provided with an iron and folic acid supplement in a highly malaria endemic region in Pemba, Zanzibar (16). In this large study, infants were provided with a multivitamin-mineral tablet with and without iron, that was dissolvable in either water or breast milk. While iron supplementation was effective for the reduction of iron deficiency and anemia in iron deficient children, it was associated with increased rates of hospitalization (primarily due to malaria and infectious disease), and mortality when given to individuals who were iron replete (with or without anemia). On advice from the Data Safety Monitoring Board, the iron-supplemented arms of the trial were discontinued after approximately 20 months. A subsequent critical review of the data from this trial led to the release of a joint statement by the WHO and UNICEF with the following recommendations:

> "Caution should be exercised in settings where the prevalence of malaria and other infectious diseases is high. Until the WHO recommendations are revised it is advised that iron and folic acid supplementation to be targeted to those who are anemic and at risk of iron deficiency. They should receive concurrent protection from malaria and other infectious diseases through prevention and effective case management. The conclusions drawn from the Zanzibar trial should not be extrapolated to fortification or food-based approaches for delivering iron, where the patterns of iron absorption and metabolism may be substantially different (3)."

The UN agencies also recommended that additional research was urgently needed to develop the most effective strategies for controlling iron deficiency and anemia in regions where malaria transmission is high.

Iron Homeostasis and infectious disease risk
The body's ability to maintain a safe equilibrium of iron is crucial and complex. Iron homeostasis is regulated at the level of absorption, unlike other minerals that are regulated through excretory mechanisms. The human gastrointestinal tract is very sensitive to iron stores and oxygen carrying capacity and has the molecular and biochemical capacity to increase (up-regulate) absorption when iron stores are becoming depleted (or hemoglobin concentration is low), and to down-regulate absorption when iron stores are replete. In an iron deficient individual, iron absorption may be as high as 40-50%, while in an iron-replete individual, iron absorption is between 5-10%. One can imagine that humans developed this sensitive and sophisticated regulatory capacity since too little iron can lead to inadequate oxygenation of vital tissues (and ultimately death), while too much iron can overwhelm the capacity of the body to safely bind the iron to protein, leaving potentially toxic free (non protein-bound) iron. In the case of malaria, it is thought that the parasite can proliferate when there is a labile pool of non protein-bound iron available. On the other hand, it has been postulated that iron deficiency might protect the host organism through increased zinc protoporphyrin in red blood cells, which inhibits parasite haemozoin formation, similar to the mechanism of certain anti-malarial drugs (17).

Potential Adverse Impact of 'Excess' Iron
Iron excess has been associated with oxidative damage in animal models, through the impact of free iron on lipid membrane peroxidation as well as DNA damage. There is a suggestion of lipid peroxidative damage from excess iron in humans, but the studies are limited in number and design. A study by Dewey et al described a degree of growth impairment in iron replete infants provided with a liquid iron supplement (18). In this study, iron supplementation was investigated in two cohorts of infants, one in Sweden and the other in Honduras. It was observed that iron supplementation (with iron syrup) of iron replete Swedish infants between the ages of 4 – 9 months demonstrated significantly decreased length and head circumference growth compared to those receiving no iron supplement. In the Honduran infants, decreased length was observed in iron replete infants between the ages of 4-6 months provided with a similar iron supplement, compared to those receiving a placebo. The results of this study were confirmed in a similar study in India, also using liquid iron supplements (19).

Overall, the findings from the above studies, as well as the one conducted in Pemba, have generated much discussion on the possible deleterious impact of iron supplementation in otherwise healthy iron-replete infants and young children. A plausible biological explanation for these observations is related to the absorption kinetics of iron provided as a supplement. With supplementation, a concentrated form of iron is provided, often in a post-prandial state, with resulting high peak serum iron concentrations (C-max) and a shorter 'time to maximum serum concentration' (T-max). It has been suggested that, under these conditions, the rate of iron absorption may be greater than the child's capacity to bind the absorbed iron (with transferrin), resulting in increased 'free iron' if only for a short period of time. As mentioned above, 'free iron' may benefit any eukaryotic pathogens concomitantly present (like malaria parasites, Yersinia, etc), with resulting proliferation of the pathogens and an adverse clinical outcome. We believe that there may be

varying outcomes associated with the form of iron and mode of delivery, leading us to ask whether the form and mode of iron delivery have an impact on the incidence of malaria among children at risk.

In the current grant, we are proposing to use a form of iron and a delivery system that differ from those used in the Pemba study in four major ways: Firstly, we plan to use a powdered mineral ‘fortificant’ as the iron source. This source differs from a typical iron supplement in a number of ways which may protect the recipient against the suggested generation of free iron. The iron source is microencapsulated ferrous fumarate. As will be discussed (below), microencapsulation protects the iron from the food matrix (thus preventing oxidation of the iron) and likely results in a lower C-max and longer T-max. Secondly, we will provide the powdered iron source for 5 months in each of the dry and wet seasons, while the Pemba study provided the supplement for a full year. Although the length of supplementation did not have an impact on the results in Pemba, results from previous studies completed by our research group have demonstrated that the use of powdered iron as a fortificant for relatively short periods of time (as short as two months) resulted in a significant increase in hemoglobin and a significant decrease in rates of anemia that lasted for as long as six months after the end of the intervention period (20). Thus we believe that a period shorter than 1 year will be sufficient to have a significant impact on anemia rates. Thirdly, although the dose of iron in the current study (12.5 mg/day) is similar to that provided to the older infants and young children in the Pemba study, as previously discussed, it will be provided in a food matrix, rather than as a supplement. We believe that this dose will be adequate to have an impact on anemia rates in children with iron deficiency, but not excessive for children whose iron stores are replete. Finally, the minerals and vitamins (including iron as microencapsulated ferrous fumarate, zinc, vitamins A, and C) will be provided in single-dose sachets (like small packets of sugar), which are easily sprinkled once daily onto any semi-solid or ‘soft’ foods. Although folic acid was included in the multimicronutrient supplement in the Pemba study, it is possible that folic acid may inhibit the action of certain anti-malarial drugs, thus it will **not** be included in the sachet. Because the powdered formulation is taken with food and due to the microencapsulation of the iron, it is likely that the absorption characteristics will be different from that of non-microencapsulated iron given in a post-prandial state – potentially reducing the amount of available free iron. **By reducing the peak labile pool of free iron, it is possible that the safety of using powdered fortificants in areas with a high incidence of infection may be increased.**

Microencapsulation
Microencapsulation is a process by which tiny parcels of a gas, liquid, or solids are packaged within a second material for the purpose of shielding the active ingredient from the surrounding environment. There can be numerous reasons for microencapsulation. These include isolation of the contents from the environment (e.g.. preventing iron from oxidizing with food), improving handling properties (e.g. preventing dangerous pesticides from coming in contact with hands) and/or controlling the release of the contents (e.g. reducing the rate of release of drugs in the gastrointestinal tract).

Effect of micoencapsulation on pharmacokinetic properties
Microencapsulation has has been shown to have an effect on the absorption characteristics of a drug by significantly reducing and delaying peak plasma concentrations (Cmax and Tmax) (21-23). Further, studies examining the effect of giving a microencapsulated drug with or without food have shown that, post-prandially, the maximum plasma concentration tends to be lower as compared to the fasted state (21, 24). Although not tested directly, the findings from these pharmacokinetic studies suggest that the use of microencapsulated iron as proposed in the current study (i.e. mixed with food) may result in a reduced Cmax (peak plasma level) and longer Tmax with a potentially reduced peak labile pool of free iron and thus a higher safety profile in a malaria endemic region.

3.2 Importance and relevance of research
Notwithstanding the debate around the adverse impact of iron deficiency anemia on malaria and possibly growth (in iron replete children), all countries, as well as the UN agencies, UNICEF and the WHO,

recognize iron as an essential nutrient and recommend a daily intake of iron between 5 -10 mg/day depending on the age of the child. It is also recommended that, whenever possible, iron should come from the diet alone; however, when dietary sources are inadequate, either supplementation or fortification is recommended. As such, the international nutrition community has been exploring ways to treat and prevent IDA through food diversification, supplementation and fortification. Unfortunately for children under age 2 years in developing countries, neither diversification nor supplementation has proven to be an effective means of coping with the problem. Although food fortification of commodities like wheat flour are used to prevent IDA in adult populations, they have not been successful for young children since the level of fortification is aimed at the adult male, and the total amount of the fortified food that is eaten is too low in the young infant or small child to meet their iron intake requirements. We and others have demonstrated over the past 10 year that 'point of use fortification' with powdered minerals and vitamins is both efficacious and effective (25-34), and this intervention is beginning to be scaled up in a number of countries where malaria is not present. To our knowledge, the safety and efficacy of providing children with iron, in the form of a powdered 'point of use' fortificant, in areas with a high prevalence of malaria has not been investigated. **We are confident that the outcome of the current proposal, combined with the results from the Pemba study, should enable more effective decision making and policy formulation regarding the use of powdered minerals and vitamins in malaria endemic regions globally**.

3.3 Significance

Overall, this investigation will provide important information on how health-related policies and programs can be improved to ensure that infants and young children in high malaria burden countries are safely protected from micronutrient malnutrition, and thus able to better achieve their growth and development potential. Specifically, the protocol will contribute the following to the literature on this topic:

1. We will determine whether the form of the iron used in the present study, and the delivery mode (powdered iron sprinkled on to food), results in an increase or decrease in malaria incidence. The Pemba study reported increased morbidity and mortality with a dissolvable iron tablet supplement which may have been rapidly absorbed from the proximal gastrointestinal tract. The form of iron to be used in the proposed study is microencapsulated ferrous fumarate, mixed in a food matrix. It is anticipated that the digestion and absorption of this form and delivery of iron is slower (lower Cmax [peak serum concentration] and higher Tmax [longer time to peak absorption]) and thus safer than a supplement.

2. As was the case in the Pemba study, we will determine whether iron replete children are more likely to have an adverse outcome, compared to iron deficient children. In the proposed protocol, we plan to enroll all otherwise healthy children without severe anemia. Based on our previous studies in Ghana, we expect that approximately 40 – 50% of children will be iron replete. Our sample size is large enough to allow for statistical evaluation of the primary outcome based on the iron status of the subjects at baseline.

It is anticipated that the proposed research will help address the confusion that has been generated regarding the safe and appropriate use of iron supplements in high malaria transmission areas. If it is demonstrated that the provision of iron, as a powder added to food, does not have an adverse effect on malaria incidence and a positive impact on anemia rates, then this type of iron delivery system can possibly be recommended for scale-up in Ghana, and in other countries with a similar malaria burden. If we demonstrate adverse effects, especially in iron replete children, then further research is needed to identify inexpensive and non-invasive methods to screen children for anemia before iron is provided. With either outcome, this investigation will provide important information on how health-related policies and programs can be improved to ensure that

infants and young children in underdeveloped countries are protected from infectious diseases and micronutrient malnutrition, and thus able to better achieve their growth and development potential.

3.4 Potential effect of these studies on the concepts, methods, treatments, services or preventative interventions that drive this field

These studies will have a major impact on interventions to treat and prevent iron deficiency anemia in malaria endemic areas. The use of powdered mineral and vitamin fortificants is being scaled-up in a number of zero or low malaria burden developing countries as a means to prevent micronutrient deficiencies, including iron deficiency. Drs. Zlotkin and Owusu-Agyei organized a one day symposium with the Ministry of Health of Ghana in 2006, including key United Nations agencies and NGOs. The government of Ghana has an Anemia Subcommittee dedicated to finding interventions to prevent anemia in Ghanaian children. The progress of the Subcommittee, however, has been markedly impaired by the results of the Pemba study and the subsequent UNICEF/WHO guidance on the use of iron in malaria endemic areas. The results of the proposed research study will provide the government of Ghana (as well as governments in other high malaria burden countries) with new and significant information for planning safe and effective anemia prevention programs.

4. PRELIMINARY STUDIES

The Research Institute, Hospital for Sick Children in Toronto and the Kintampo Health Research Centre in Ghana have a long standing and collaborative working relationship. This provides the proposed project with a well-established and unique team of expertise in global health generally, and specifically in the area of iron deficiency anemia, malaria control and research (clinical trials) methodology.

Both Drs. Owusu-Agyei at the Kintampo Health Research Centre (KHRC) and Zlotkin (Research Institute, Hospital for Sick Children and University of Toronto) have extensive experience as researchers in the fields of malaria and in infant and young child nutrition. Zlotkin, a professor of Pediatrics, Nutritional Sciences and Public Health Sciences at the University of Toronto has worked collaboratively with the Kintampo Health Research Centre since the late 1990s, originally with Dr. Paul Arthur, Director at the time (now passed away) and for the past six years with Dr. Owusu-Agyei who has been the Director of KHRC since 2002. Zlotkin's original efficacy trials on 'Sprinkles' (a powdered mineral and vitamin fortificant) were completed at KHRC. The first collaborative manuscript was published in 2002 (29) the most recent publications from Ghana were published with Dr. Owusu-Agyei in 2006 (30, 35).

Sprinkles are sachets (like small packets of sugar) containing a blend of vitamins and minerals in powder form, which are easily sprinkled onto different foods. The single-serving sachets enable families without access to commercially fortified foods to add essential vitamins and minerals directly to traditional foods prepared in the home. They are inexpensive to produce, have no special storage requirements and are simple to use, even by those who cannot read. Because the iron is microencapsulated, there is no staining of a young child's teeth as may be the case with iron syrup or drops. A major advantage of the 'sprinkles' concept is that local foods can continue to be used and, thus, there is no need to teach caregivers how to prepare new and often expensive store-bought foods. Sprinkles were invented by the PI, Dr. Stanley Zlotkin, who, over the past ten years, has led a collaborative research team in multiple countries globally. Together these collaborative teams have demonstrated the absorption, efficacy, and effectiveness of microencapsulated iron, as well as the acceptability of the sprinkles concept (powdered minerals and vitamins) to treat and prevent iron and other micronutrient deficiencies (28, 29, 31, 35, 36).

As previously mentioned, the first randomized controlled trials were conducted in Ghana at KHRC. A total of 5 studies were completed at KHRC and published in peer-reviewed journals, including the American Journal of Clinical Nutrition and the Journal of Nutrition. More recently Dr. Zlotkin has collaborated with a research team from the University of California at Davis (led by Dr. Kay Dewey), and partners at the University of Ghana at

Legon, to examine the relative merits of a powdered mineral and vitamin fortificant versus a fortified spread for the treatment and prevention of micronutrient deficiencies. Two recent publications describe that work (37, 38).

As well as having worked in Ghana with KHRC, Dr. Zlotkin has experience with a number of other countries and research organizations. He has a long-standing research collaboration with the Research and Evaluation Division of BRAC in Bangladesh. He and his collaborators at BRAC have evaluated the effectiveness and acceptability of powdered mineral and vitamin preparations when provided on a weekly basis (versus daily) and with a flexible regimen (20, 39). Dr. Zlotkin has also had successful research collaborations with the Chinese Centre for Disease Control (40), the Swiss Red Cross in Kyrgyzstan, World Vision International in Mongolia, CARE International in Benin (41), Agha Khan University in Pakistan (32, 42) and the King Edward Medical Hospital in India (25, 33, 43). In addition, he has collaborated with health economists to assess the cost effectiveness of ‘point of use’ powdered mineral and vitamin products (44). In northern Canada, powdered minerals and vitamins were examined in ‘First Nations’ populations (45-47). Powdered minerals and vitamins have also been used, in collaboration with the World Food Program, World health Organization and Helen Keller International in relief situations (48).

Dr. Stanley Zlotkin has beneficial interests in certain intellectual property rights to his invention known as "Sprinkles". These interests include (i) patent rights for the United States and Canada only, which are held by Ped-Med Limited, a Canadian corporation, of which Dr. Zlotkin is the sole shareholder; and (ii) trade-marks rights in various jurisdictions to the name "Sprinkles" which are held either by Ped-Med Limited, or by the Sprinkles Global Health Initiative Inc. a Canadian not-for-profit corporation of which Dr. Zlotkin is a member.

Global recognition of a ‘powdered mineral and vitamin fortificant’ as an important, new tool for improving the health of children

The concept of using powdered mineral and vitamins packaged in a single-serving package has been recognized by international agencies such as UNICEF, the World Health Organization (WHO) and the World Food Program (WFP). Its success has been widely reported in both academic journals and the popular press. A number of government ministries of health have enacted legislation to include home fortification with powdered mineral and vitamin fortificants in recently updated national nutrition policies (Mongolia, Bolivia, Bangladesh, etc)

About the Kintampo Health Research Centre

Kintampo Health Research Centre (KHRC) is a well-established, African-based, research centre. The mission of KHRC is to conduct public health research and develop health research capacity which will contribute to a significant reduction in ill-health and the achievement of the Millennium Development Goals for Africa’s most disadvantaged communities. The African identity of KHRC is important as it emphasizes African solutions to African health challenges. KHRC is one of three field research centers of the Health Research Unit of Ghana Health Service established in 1994. KHRC is situated in the middle belt of Ghana in the Brong Ahafo Region with a mandate to serve health policy and practice throughout Ghana and Africa.

There are over 500 employees at KHRC. KHRC has over 12 years of extensive experience in health research in Ghana. Among many areas of activity in which it has demonstrated skill, knowledge and core competency it has developed one of the largest and most reliable district surveillance systems (DSS) and study populations in Africa. KHRC has an established reputation for quality research and personnel. KHRC is highly regarded and is the preferred partner of governments, organizations and donors in health research initiatives in particular large-scale health research trials. KHRC funding collaborators include government, bilateral and multilateral institutions, private corporations, private charities and international organizations from Africa, Europe, Canada and the USA

KHRC Priority Research Areas

- Communicable diseases (CDs), particularly Malaria, TB and HIV/AIDS
- Sexual and Reproductive Health
- Maternal, Neonatal and Child Health
- Mental Health
- Non-communicable diseases (NCDs) such as hypertension and cancer
- Health Systems
- Using the DSS to track progress towards the Millennium Development Goals (MDGs) using indicators such as mortality levels, patterns and trends

Since 2003, Dr Owusu-Agyei and his research teams at KHRC have characterized the epidemiology of malaria in the Kintampo area in the middle belt of Ghana (49-51). He has documented the incidence of malaria in children less than 5 years in this area as 7 attacks per child per year (Owusu-Agyei et al., in press). The transmission levels have been high throughout the year with an average inoculation rate of 269 infective bites per person per year (Owusu-Agyei et al., in press). Dr Owusu-Agyei and his team of researchers are documenting the manifestations (both clinical and laboratory indicators) of severe and complicated malaria. Several antimalarials have been tested and evaluated in this area (52) and currently the most advanced malaria vaccine is being tested in Kintampo as one of several African countries. For more details on the KHRC laboratory and clinical facilities, please see pages 22-23 of this application (Resources Format Page).

The Kintampo Health Research Centre (KHRC) was awarded the 2008 Prince of Asturias Award for International Cooperation in recognition of its contribution to the fight against malaria in sub-Saharan Africa. The Prince of Asturias Foundation was formed by His Royal Highness, the Prince of Asturias, heir to the throne of Spain. It has conferred its awards since 1981. The awards are intended to acknowledge scientific, technical, cultural, social and humanitarian work carried out internationally by individuals, groups or in the categories of communication and humanities, social sciences, arts, letters, scientific and technical research, international cooperation, concord and sports.

The collaboration between the Research Institute at the Hospital for Sick Children in Toronto and KHRC in Ghana provides this project with a well-established and highly experienced team of experts in iron deficiency anemia, malaria control and Sprinkles-based research methodology. Given the past working relationship between the two partners, the protocol can be put into action without the need for preliminary relationship and trust-building activities between the two organizations.

Global Health at the University of Toronto, the Hospital for Sick Children and its Research Institute
One of the strategic directions of the Hospital for Sick Children in Toronto is “to lead nationally and internationally”. Operationally this strategic goal is being met through programs within the Hospital, its Research Institute and its affiliation with the University of Toronto. For example, the Program for Global Pediatric Research at the Hospital was formed to address the disparity between the scientific research resources available in high-income countries and the quantity of scientific research focused on the health of children in mid- and low-income countries. This Program works at the centre of a global network to inform, educate, and facilitate international research cooperation and collaboration, as well as advocate for research to improve the health of all children. Zlotkin is a member of that program.

The Research Institute at The Hospital for Sick Children undertakes child-centered research across the life continuum from fetal origins to adult outcomes, including fundamental discovery, applied research, and outcomes and impact. Support for clinical trials at the Research Institute is through the Clinical Research Support Unit (CRSU). This Unit is a consultation service operated by the Child Health Evaluative Sciences Research Program of the Research Institute. The mandate of the CRSU is to improve the quality of clinical

research at the Hospital by providing consultation in the areas of study design and methodology, statistical analysis, and data management.

Dr. Zlotkin is Head of the Division of Gastroenterology, Hepatology and Nutrition at the Hospital for Sick Children, a Senior Scientist in the Research Institute in the Child Health and Evaluative Sciences Research Program, and a Professor (of Pediatrics, Nutritional Sciences and Public health Sciences) at the University of Toronto. As a Professor, he has full access to the research facilities at the University. The University of Toronto is the largest university in Canada with more than 50,000 undergraduate students and 10,000 graduate students. It has more than 18,000 staff and faculty in 520 graduate programs and 42 professional programs. Its library system has over 18 million holdings, one of the top five research libraries in North America and its research grant and contract support is over $800 million.

The combined infrastructure of the Hospital, the Research Institute and the University of Toronto provide more than ample infrastructure support for the collaborative project described in this application.

5. RESEARCH DESIGN AND METHODS

5.1 Design Conceptual or Clinical Framework

Study Design

The proposed study is a community-based blinded randomized controlled trial with 2 study arms that will be conducted in two phases:

- Phase I will take place during the dry season (December to April), when malaria transmission rates are lower. Eligible subjects (one per household) will be individually randomized to receive a daily dose of either a powdered vitamin/mineral fortificant containing 12.5 mg of iron (plus ascorbic acid, vitamin A and zinc), or a placebo (containing all micronutrients excluding iron), added to complementary foods, for 5 months.

- Phase II will take place during the wet season (June to October), when malaria transmission rates are higher. Eligible subjects, who did not participate in Phase I, will be individually randomized to one of the two study arms as described above and followed for 5 months.

A dual phase design, with two unique cohorts, was chosen so that preliminary results (at the end of phase 1) could be assessed by an independent Data Safety and Monitoring Committee. It is possible that during the dry season no impact of iron will be detected, while during the wet season, an impact will be observed. With this possible outcome, it is potentially feasible to translate this knowledge into a Ministry of Health Program to only provide iron supplementation (fortification) during the dry months of the year (December to April).

Primary and Secondary Outcomes

The primary outcome will be ***clinical malaria***, defined (according to WHO criteria) as parasitemia (malaria parasites detected on a blood smear) of any density plus history of fever (within 48 hours) or axillary temperature >37.5^{0}C. Criteria for clinical malaria will be taken from the UNICEF Case Management Series, entitled, “Promoting Rational Use of Drugs and Correct Case Management in Basic Health Services – Malaria Prevention and Treatment” published by UNICEF’s Programme Division in cooperation with the World Health Organization in 2000. To distinguish unique malaria episodes, treatment will be supervised and follow-up blood smears will be collected from the child as described below in Section 5.2(c).

Secondary outcomes will include: (i) changes in anemia status; (ii) the severity of the clinical malaria (based on level of parasitemia); (iii) cerebral malaria; (iv) hospitalization (from any cause) (v) death; (vi) pneumonia; (vii) diarrhea and dehydration.

Subjects/study population
Infants and young children (6-24 months of age), living in the Brong Ahafo Region of northern Ghana, will be included in the study if, at baseline, they are ingesting weaning foods in addition to breastmilk; free from malaria or other major illness; afebrile; living in the study area for the duration of the intervention and follow-up period; and if parental consent is obtained. Inclusion of a child into the study will proceed after the possible risks and benefits are discussed with the parents (in the appropriate local language), and a signed consent is obtained. The exclusion criteria are as follows: severe anemia (hemoglobin <70g/L), weight-for-height <-3 z-score (severe wasting), kwashiorkor (defined as evidence of edema), congenital abnormality, treatment with iron supplements in the past 6 months, or any chronic illness. Children that are severely anemic (Hb<70 g/L) and/or have symptoms of clinical malaria will be excluded from entry to the study and referred to the local health provider for treatment according to Ghana Ministry of Health guidelines.

Sample Size
We hypothesize that the incidence of malaria will be significantly higher in children receiving iron versus a placebo of micronutrients without iron. It was decided not to use the change in anemia status as a primary outcome since, in all previous studies with powdered mineral and vitamin fortificants, anemia rates declined compared to placebo controls, or were no different from the improvement in anemia rates observed with iron drops.

Based on the 2006 estimate of "cases and deaths from fevers suspected of being malaria" for children under 5 years of age in all of Ghana (53), the baseline rate of 3.44 episodes/child/year was assumed. We estimated that a total of 351 person-years would be required to detect a 15% increase in malaria incidence rates with 80% power and at a 5% type I error. Based on the experience and expertise of the PI and co-investigator, a difference of 15% in malaria incidence was considered to be clinically significant. Knowing that all children enrolled in the trial will begin the 5-month intervention period at a similar risk level, and accounting for 15% loss to follow-up, as well as the need to test the hypothesis twice (due to the interim analysis by the Data Safety and Monitoring committee), the sample size has been calculated as 1940 children per seasonal cohort (970 per group), or a total of 3880 subjects (see Table 1 below). All calculations were reviewed by 2 statisticians who will be involved in subsequent analyses.

Table 1: Sample size calculation adjusted for testing the hypothesis twice

Least Meaningful Difference	2006 rates	Adjustment for use of bed-net	Rate unexposed	Rate exposed	Person-years	Follow-up years	n	Loss to follow-up	Sample size per group*
0.05	3.44	0.75	2.58	2.71	3017	0.42	7240	0.15	8326
0.1	3.44	0.75	2.58	2.84	772	0.42	1854	0.15	2132
**0.15**	**3.44**	**0.75**	**2.58**	**2.97**	**351**	**0.42**	**843**	**0.15**	**970**
0.2	3.44	0.75	2.58	3.10	202	0.42	485	0.15	558

*Adjusted for estimated bed net use and loss to follow-up

5.2 Procedures
Recruitment, Randomization Process and Blinding
Subjects will be recruited from villages in the Kintampo Health Research Centre (KHRC) study area once permission has been obtained from the village assemblies and elders, as well as the parent or guardian of the eligible child. Field researchers, employed by the KHRC, will visit individual households/compounds and interview parents or caregivers regarding the inclusion criteria. Once consent has been obtained and documented via signature or thumbprint, eligible children (one per household/compound) will be enrolled and randomized, using a computer-generated model, to either the iron (Fe) or placebo (P) group.

Sachets containing the powdered minerals with and without iron will look and taste identical with the exception that each package will be marked with an ‘A’ or ‘B’ denoting packages with or without iron. The study team and caretakers of children will be blinded to the ‘A’ or ‘B’ designation. Only the manufacturer of the powdered fortificant (in Ghana) and a research pharmacist each in Toronto and Kintampo will hold the key to the randomization and ‘sachet’ code lists. The key will be revealed to the ‘Data and Safety Monitoring Committee (DSMC)’ at the end of the first phase if a significant difference in outcomes is observed, and to the researchers after the database is closed and statistical analyses completed.

Supply and content of the powdered mineral and vitamin fortificant.
The applicant (SZ) has extensive experience with the powdered mineral and vitamin product both in research protocol and in working with governments, UN agencies and NGOs for scaling up the intervention. The product will be procured from a production facility in Canada, India or Bangladesh. Each of these facilities has supplied a reliable high quality product to the applicant for past research projects. The production facilities in India and Bangladesh are UNICEF approved facilities. The dose of micronutrients including iron is shown in Table 2 below. The dose of individual nutrients is based on WHO or IOM-DRI guidelines, or estimates based on these guidelines.

Table 2: Justification of a powdered mineral and vitamin supplement dose for infants and young children

	6-11 mo		12-24 mo		***Powdered fortificant***
	WHO[1]	IOM DRI[2]	WHO	IOM DRI	
Vitamin A, µg RE	400	500*	400	300	400
Vitamin C, mg	30	50*	30	15	30
Folic Acid, µg	80	80*	160	150	OMIT
Iron, mg[3]	9.3	11	5.8	7	12.5
Zinc mg[4]	4.1	3	1.1	3	5

[1] Recommended Nutrient Intakes. Source: Joint FAO/WHO Expert Consultation. (2002) Vitamin and mineral requirements in human nutrition. World Health Organization, Geneva, Switzerland.
[2] Recommended Dietary Allowances (RDA). Sources: Institute of Medicine, Dietary Reference Intakes. National Academy Press, Washington D.C.
[3] The dose of 12.5 mg is the current INACG/WHO/UNICEF recommendation for children 6-24 months of age for large scale distribution. It assumes a medium bioavailability (5-10%).
[4] Assuming moderate bioavailability (30%)
* Based on Adequate Intake (AI) estimates

Clinical protocol
a) *Baseline:* After consent is provided, subjects will be screened for anemia (based on hemoglobin) and parasitemia. Enough blood will be taken to determine ferritin (SF), C-reactive protein (CRP), serum transferrin receptor (TfR) and zinc protoporphyrin (ZnPP). Those who are severely anemic (Hb <70 g/L), or have clinical signs of infection (including malaria) will be referred to the study clinician who will offer the child the standard care, as defined by the Ghana Ministry of Health. All other children will be enrolled and randomized to either the iron (Fe) or placebo (P) group. The child’s weight and length measurements will be recorded, as well as any relevant demographic (age, gender) and historical health information (birth weight, immunization status, and previous hospitalizations). Caregivers will also be asked to provide information regarding their usual feeding practices and health-seeking behaviours. All households will be provided with a treated bed net and a supply of powdered fortificant with oral instructions on how to use each.

b) Monitoring visits: Field researchers will visit each subject at their home every week in the first month of the study and at 4 week intervals for the next 4 months. At each visit, the field researcher will conduct a health assessment (including axillary temperature) and collect information on feeding history, supplement compliance, bed net use and morbidity. At the initial weekly visits, 7 new sachets will be provided, and then at the start of the second month, 30 new sachets will be provided to last for a month. Clear instructions for storage and administration of sachets will be provided to families. All used and unused sachets will be counted and recorded. It has been our experience in other similar studies in Ghana and elsewhere that by providing pre-emptive information on the impact of the fortificant to parents (like telling parents that stools will darken from the iron, and that children will become more active and have enhanced appetites), the adherence to the use of the sachets is generally in the range of 70 – 98%. We will provide this pre-emptive information in the current study.

(c) Tracking the subjects: All subjects who become ill and need to visit a health facility will be identified and tracked through their study identity cards. The use of such cards is standard practice at the Kintampo Health Research Centre (KHRC). If a fever has been reported or recorded, a blood sample will be taken to determine parasite species and count. If the child is admitted to the hospital or health centre, blood parasite level will similarly be determined, and further tests will be conducted (as needed) to rule out cerebral malaria, pneumonia, diarrhea, and/or dehydration. All treatments will follow the treatment guidelines in Ghana (Ghana Ministry of Health). For those assessed to have malaria, the first-line antimalarial (Artesunate-Amodiaquine) will be prescribed and provided. To determine if the treatment has been successful, and from the perspective of the study, to distinguish unique malaria episodes, treatment will be supervised and follow-up blood smears will be collected from the child on the 3rd, 7th, 14th and 28th day following treatment (this process has been successfully used in past trials including “A multi-centre, randomized, double-blind, double dummy study comparing the efficacy and safety of chlorproguanil-dapsone-artesunate versus artemether-lumefantrine in the treatment of acute uncomplicated *Plasmodium falciparum* malaria in children and adolescents in Africa Protocol #: SB-714703/005. Collaborations among KHRC/LSHTM/GSK/MMV/WHO”). All slides (with thick and thin films) will be stained with Giemsa following fixing of the thin film and read twice by independent microscopists blinded to each other’s reading. A third microscopist will be asked to read the slide if there is disagreement between the first two. The readings will be used to determine if the treatment has been successful or not.

c) Follow up: At the end of 5 months, subjects will again be screened for anemia, ferritin, CRP, serum transferrin receptor, zinc protoporphyrin and a blood smear for their malaria status. The subjects will be weighed and measured, and caregivers will again be asked about feeding history, supplement use, and bed net use. Any remaining sachets will be collected and counted. All families that have completed the intervention will be provided with a study closure package (including some rice and soap) as has been the standard goodwill practice of KHRC to participants from the communities. For those children who are anemic, based on their end-line hemoglobin assessment, an additional 60 sachets of iron-containing powdered fortificant will be provided. Providing iron to anemic children is not a safety issue in this case because they will be eligible for treatment according to the WHO statement on the use of iron in malaria endemic areas (3).

d) *Discontinuation of supplementation*. Supplementation will be stopped if any of the following is reported: admission to the hospital ward for >5 days; evidence of chronic diarrhea (defined as loose stools lasting >4 weeks); prolonged fever of unknown origin; emergence of any exclusion criteria after randomization. Prompt, daily disclosure by field workers of any concerns will be encouraged. If necessary, children will be referred to local physicians for treatment according to local standards. Reasons for stopping supplementation voluntarily will be recorded.

5.3 Analyses

Laboratory and Clinical Analyses
All laboratory analyses will be conducted at the Kintampo Health Research Centre by qualified laboratory technicians.

Screening for malaria will be performed using RDTs to help decide on treatment and blood smears for microscopy to provide counts. All clinical signs and symptoms will be interpreted using standardized ‘case definitions’ as described in previous studies (16, 54). For example, cerebral malaria (defined by a parasite count >5000/μL blood and a concurrent score of $\leq$2 on the Blantyre coma scale, with or without convulsions), pneumonia (defined by the presence of a cough or breathing difficulties, tachypnea, lower chest wall indrawing, and the appearance of consolidation or pleural effusion on a chest X-ray), diarrhea (defined by $\geq$3 loose or watery stools in the previous 24 hours), and dehydration (defined by lethargy, sunken eyes, and decreased skin turgor [>2 seconds for skin to return following a skin pinch]) will be assessed in the hospital or health centre using standard procedures/methods. Severe malaria disease will be diagnosed based on symptoms and signs occurring at presentation or developing during admission according to generally accepted case definitions as have been used in all previous studies in Ghana. These ‘case definitions’ are available on request.

For identifying and analyzing cause-specific deaths or admissions to hospital, we will use exclusive categories to ensure that independent events are not classified more than once. As such, we will first allocate malaria related causes, then pneumonia and other infection related causes, and finally diarrhea and others.

Blood samples will be assayed for hemoglobin (Hb) using Hemocues (Mallinckrodt, USA). Plasma ferritin, CRP, zinc protoporphyrin and transferrin receptor will be assayed using standard methods, including internal and external reference standards. Since CRP is a measure of inflammation, blood samples from subjects with elevated CRP values (>8 mg/L) will be excluded from further analysis (55). It should be noted however, that even in a malaria endemic area, albeit in school children (in Zanzibar), iron status assessment using these indicators may not be seriously influenced by malarial infection (4). Zinc protoporphyrin is a metabolic intermediate of the hemoglobin synthetic pathway which accumulates in red blood cells when iron supply is limited. It can be easily measured fluorometrically and is expressed as a ratio to heme (ZPP/H). In adults, ZPP/H correlates inversely with plasma ferritin across a wide range of ferritin concentrations (56), and is inversely related to the amount of stainable iron in the marrow (57). In adults (56, 58, 59) and children (60) ZPP/H has been shown to be more sensitive than the packed cell volume or hemoglobin concentration in detecting iron deficiency. It is particularly suited as a screening test because it is cheap, convenient (61) and can be carried out on a single drop of blood (62). ZPP/H ratios are expressed as μg/g hemoglobin (Hb) and plasma ferritin as μg/L. ZPP/H and plasma ferritin will be log transformed (to the base 10) before analysis to normalize the distribution, as both are usually positively skewed.

TfR and ZnPP are sensitive measures of iron-deficient erythropoiesis and have been used to define iron status in children in developing countries (63-67). TfR may have an advantage over SF because it is unaffected by the acute phase response (63, 68, 69). However, the specificity of TfR may be low because it can be increased by malaria (70), megaloblastic anemia due to vitamin deficiencies (71), and hemoglobinopathies such as sickle cell disease (72), hemoglobin H disease, and the thalassemias (73, 74). ZnPP has advantages of low cost and simplicity, but its specificity may be low as it also can be increased by malaria and other infections, chronic inflammation, and hemoglobinopathies (60, 63, 64, 75-77). To improve specificity, SF is often combined with TfR, ZnPP, or both (78). In the current protocol, we will include all three measurements in our assessment of iron status.

Zimmerman et al recently studied the use of serum transferrin receptor and zinc protoporphyrin as indicators of iron status in African children (Côte d’Ivoire). He determined the most sensitive and specific markers for iron status and their ‘optimal’ diagnostic cutoffs (TfR >9.4 mg/L or ZnPP > 52 µmol/mol heme) after correcting for

inflammation (79). We plan to use these cutoffs to define iron status as iron replete and anemic, iron deficient and anemic, of iron replete and not anemic. Zimmerman defined anemia as a hemoglobin concentration below the WHO cutoff values (110 g/L) minus 10 g/L, and normal ferritin as > 30 µg/L (79). Several investigators have argued that a SF concentration >30 µg/L should be used to define adequate iron stores in developing countries with a high prevalence of infection (63, 80). Therefore, we have chosen a SF cutoff of >30 µg/L as being indicative of iron sufficiency.

To summarize: anemia will be defined as Hb <100 g/L; IDA will be defined as Hb < 100 g/L plus one of low ferritin (<30 µg/L), high TfR (>9.4 mg/L) or ZnPP (>52 µmol/mol heme); and iron deficiency will be defined as one of low ferritin (<30 µg/L), high TfR (>9.4 mg/L) or ZnPP (>52 µmol/mol heme). We recognize that infection and inflammation will confound the interpretation of SF, TfR, and ZnPP; thus we will exclude and/or adjust these indicators for those children who have signs of infection or inflammation, as defined by an elevated CRP concentration (>8 mg/L) (55, 81).

5.4 Data Collection and Management
Baseline demographic, anthropometric, and health history data, as well as information pertaining to feeding practices and health-seeking behaviours, will be collected by field researchers employed by the KHRC. All blood samples will be collected by qualified individuals, trained specifically in pediatric phlebotomy techniques.

Visual Basic will be used to manage data. All data obtained in the field will be entered by the end of the next day. The systems used will have extensive range and checking facilities. Possible errors will be verified with field or hospital staff on a daily basis. Data collection and supplement allocation will be rigorously controlled with the help of computer monitoring. For all outcomes, a double-data entry will be used to detect errors. Flow of information, distribution of supplements, and collection of samples between households and villages and the central office at the Kintampo Health Research Centre will be ensured by supervisors on motorbikes on the same day.

5.5 Data Analysis
All variables will be first explored and summarized using descriptive statistics such as number of events in person-time, incidence rates, means and standard deviations, medians and ranges, counts and proportions, and various graphs, as appropriate.

Primary analysis
The primary outcome, incidence of malaria, will be compared between the two groups using Poisson regression. A 95% confidence interval for the ratio between the incidence in the iron group and the incidence in the placebo group will be calculated. The upper boundary of this confidence interval will be compared to the 10% tolerance limit. If it is lower, then non-inferiority can be concluded.

Secondary analysis
Univariate Poisson regression with the same outcome will be used to evaluate associations with other variables: iron status at baseline, breast-feeding, age, gender and use of bed-net. These will then be introduced and re-evaluated in a multiple Poisson regression model, which will allow for adjusted group comparisons. Most importantly, by including the baseline iron status variable and the interaction with the main group variable, we can focus on the iron sufficient children that are receiving iron supplements and compare them against the others. The Poisson models will be validated by checking the model fit and for over-dispersion. Adjustments or transformations will be used if necessary.

The number of hospital admissions will be analyzed in a similar fashion, using Poisson regression. The parasite count outcome will be compared between the two groups, using a t-test. Univariate associations with other factors will be verified here as well, using t-tests, correlation coefficients and univariate regression.

Ultimately a multiple regression model will be developed, again for the purpose of adjusted comparisons between groups.

The normality of the data will be verified and, in the case of any departures, a log-transformation will be used and the data reanalyzed. If the log-transformation fails to achieve normality, a nonparametric alternative will be used instead. All statistical analyses will be carried out using SAS 9.1. The Analysis will be carried out on an intention-to-treat basis including all randomized children.

Upon the completion of the first intervention phase, an interim analysis will be performed by a Data and Safety Monitoring Committee (DSMC) as described above (primary analysis). The study will be stopped if the incidence of malaria is higher in one group.

5.6 Data Interpretation
Findings from the proposed research will provide evidence regarding the safety and efficacy of providing daily iron supplementation (12.5 mg/day), in the form of a micronutrient powder that is added to complementary foods, during the dry and wet season in a malaria endemic area.

5.7 Data Sharing Plan
Findings from the proposed research will be shared according to the applicable NIH policy for foreign institutions.

5.8 Potential Difficulties and Limitations
a) Blinding: Although research staff will be blinded to the intervention, the stools of children receiving the iron containing fortificant will likely be darker (blacker) than those receiving the placebo. The applicants are unable to control for this likelihood.

b) If after the first phase (the dry season) there is more clinical malaria or more severe malaria or health complications in the group receiving the iron-containing fortificant, the DSMC may recommend terminating the study before the second phase begins.

c) It will not be possible to compare the results of the proposed current study to many of the results from the Pemba study. In Pemba, the primary outcome was mortality, which necessitated a sample size of more than 30,000 subjects. Also, in the current protocol, folic acid will not be included in the powdered mineral and vitamin supplement. Based on the estimated dietary folic acid intake of older infants and children in West Africa, folic acid deficiency should not be a problem (fruit is plentiful and inexpensive). Lastly, in the current protocol all children will receive and be encouraged to use bed-nets. This was not the case in the Pemba study.

d) Although studies in adults have suggested that elevations in TfR (82) or in ZnPP (75) can be definitive indicators of iron deficiency, there is large overlap in the distributions of these indicators in children with iron deficiency anemia as well as those with normal iron status. This overlap may be explained by a greater variability in the erythroid mass in children than in adults (83) together with the many variables affecting children in developing countries that influence TfR and ZnPP independent of iron status. Because of this overlap, the sensitivity and specificity of TfR and ZnPP in identifying iron deficiency and IDA may not be as high as we would prefer regardless of the diagnostic cutoffs chosen. Despite this limitation. all three biomarkers of iron status, ferritin, TfR and ZnPP, will be statistically evaluated individually and in groupings since this measure is very important in the context of the current study.

5.9 Tentative Project Timetable

Tasks	Location	Duration (weeks)	Jun 2009	Sep 2009	Oct 2009	Nov 2009	Dec 2009	Mar 2010	Apr 2010	May 2010	Jun 2010	Oct 2010	Nov 2010	Dec 2010	Jan 2011	Feb 2011	Mar 2011	Apr 2011	May 2011
Ethics approval	Toronto/Ghana	12																	
Procurement of supplies	Toronto/Ghana	12																	
Data management setup	Toronto/Ghana	12																	
Shipping	Toronto/Ghana	12																	
Development of training materials	Ghana	12																	
Training of staff	Ghana	12																	
Data management setup	Ghana	12																	
Clearing of supplies	Ghana	12																	
Recruitment of subjects for first cohort	Ghana	8																	
**Complete Phase 1**	Ghana	22																	
Biochemical analyses	Ghana	8																	
Cleaning and compiling data (primary outcome) for DSMC assessment	Toronto/Ghana	2																	
DSMC assessment	Toronto/Ghana	2																	
Preparation Phase 2	Toronto/Ghana	2																	
Recruitment of subjects for second cohort	Ghana	8																	
**Complete Phase 2**	Ghana	22																	
Biochemical analyses	Ghana	8																	
Cleaning and compiling data (primary and secondary outcomes) from Phases 1 and 2	Ghana	8																	
Data analysis	Toronto/Ghana	4																	
Manuscript writing and submission	Toronto/Ghana	8																	

The timing of this study is complicated by the need to enroll and study subjects during two distinct seasons, the dry season (December to April) and the wet season (June to October). Because of the need to have the 'data and safety monitoring committee (DSMC) ' review the outcomes between seasons, we will ensure that the data sets are complete and 'clean' by mid May, leaving the DSMC two weeks to complete their analysis. Note that some periods overlap. For example, the biochemical analysis will take place during and at the end of the trial.

7. BIBLIOGRAPHY AND REFERENCES

1. McCann JC, Ames BN. An overview of evidence for a causal relation between iron deficiency during development and deficits in cognitive or behavioral function. Am J Clin Nutr 2007;85(4):931-45.
2. Bhagwati J, Bourguignon F, Kydland F, Mundell R, North D, Schelling T, et al. Copenhagen Consensus 2008 Frederiksberg, Denmark: Copenhagen Concensus Centre; 2008.
3. WHO/UNICEF. Joint statement: Iron supplementation of young children in regions where malaria transmission is intense and infectious disease highly prevalent; 2006.
4. Stoltzfus RJ, Kvalsvig JD, Chwaya HM, Montresor A, Albonico M, Tielsch JM, et al. Effects of iron supplementation and anthelmintic treatment on motor and language development of preschool children in Zanzibar: double blind, placebo controlled study. Bmj 2001;323(7326):1389-93.
5. Grantham-McGregor S, Ani C. A review of studies on the effect of iron deficiency on cognitive development in children. J Nutr 2001;131(2S-2):649S-666S; discussion 666S-668S.

6. Bates CJ, Powers HJ, Lamb WH, Gelman W, Webb E. Effect of supplementary vitamins and iron on malaria indices in rural Gambian children. Trans R Soc Trop Med Hyg 1987;81(2):286-91.
7. Smith AW, Hendrickse RG, Harrison C, Hayes RJ, Greenwood BM. The effects on malaria of treatment of iron-deficiency anaemia with oral iron in Gambian children. Ann Trop Paediatr 1989;9(1):17-23.
8. Oppenheimer SJ. Iron and its relation to immunity and infectious disease. J Nutr 2001;131(2S-2):616S-633S; discussion 633S-635S.
9. van den Hombergh J, Dalderop E, Smit Y. Does iron therapy benefit children with severe malaria-associated anaemia? A clinical trial with 12 weeks supplementation of oral iron in young children from the Turiani Division, Tanzania. J Trop Pediatr 1996;42(4):220-7.
10. Gera T, Sachdev HP. Effect of iron supplementation on incidence of infectious illness in children: systematic review. Bmj 2002;325(7373):1142.
11. Atkinson SH, Rockett K, Sirugo G, Bejon PA, Fulford A, O'Connell MA, et al. Seasonal childhood anaemia in West Africa is associated with the haptoglobin 2-2 genotype. PLoS Med 2006;3(5):e172.
12. WHO/UNICEF. World Malaria Report 2005. In. Geneva: http://www.rbm.who.int/wmr2005/

13. WHO. World Health Report 2003: Shaping the future; 2003.
14. Appawu M, Owusu-Agyei S, Dadzie S, Asoala V, Anto F, Koram K, et al. Malaria transmission dynamics at a site in northern Ghana proposed for testing malaria vaccines. Trop Med Int Health 2004;9(1):164-70.
15. Koram KA, Owusu-Agyei S, Fryauff DJ, Anto F, Atuguba F, Hodgson A, et al. Seasonal profiles of malaria infection, anaemia, and bednet use among age groups and communities in northern Ghana. Trop Med Int Health 2003;8(9):793-802.
16. Sazawal S, Black RE, Ramsan M, Chwaya HM, Stoltzfus RJ, Dutta A, et al. Effects of routine prophylactic supplementation with iron and folic acid on admission to hospital and mortality in preschool children in a high malaria transmission setting: community-based, randomised, placebo-controlled trial. Lancet 2006;367(9505):133-43.
17. Iyer JK, Shi L, Shankar AH, Sullivan DJ, Jr. Zinc protoporphyrin IX binds heme crystals to inhibit the process of crystallization in Plasmodium falciparum. Mol Med 2003;9(5-8):175-82.
18. Dewey KG, Domellof M, Cohen RJ, Landa Rivera L, Hernell O, Lonnerdal B. Iron supplementation affects growth and morbidity of breast-fed infants: results of a randomized trial in Sweden and Honduras. J Nutr 2002;132(11):3249-55.
19. Majumdar I, Paul P, Talib VH, Ranga S. The effect of iron therapy on the growth of iron-replete and iron-deplete children. J Trop Pediatr 2003;49(2):84-8.
20. Ip H, Hyder SM, Haseen F, Rahman M, Zlotkin SH. Improved adherence and anaemia cure rates with flexible administration of micronutrient Sprinkles: a new public health approach to anaemia control. Eur J Clin Nutr 2007.
21. Bergdahl B, Bogentoft C, Jonsson UE, Magnusson JO. Fasting and postprandial absorption of digoxin from a microencapsulated formulation. Eur J Clin Pharmacol 1983;25(2):207-10.
22. Olver JS, Burrows GD, Norman TR. The treatment of depression with different formulations of venlafaxine: a comparative analysis. Hum Psychopharmacol 2004;19(1):9-16.
23. Baldi A, Bontempo V, Cheli F, Carli S, Sgoifo Rossi C, Dell'Orto V. Relative bioavailability of vitamin E in dairy cows following intraruminal administration of three different preparations of DL-alpha-tocopheryl acetate. Vet Res 1997;28(6):517-24.
24. Lakritz J, Wilson WD, Marsh AE, Mihalyi JE. Effects of prior feeding on pharmacokinetics and estimated bioavailability after oral administration of a single dose of microencapsulated erythromycin base in healthy foals. Am J Vet Res 2000;61(9):1011-5.
25. Zlotkin SH, Christofides AL, Hyder SM, Schauer CS, Tondeur MC, Sharieff W. Controlling iron deficiency anemia through the use of home-fortified complementary foods. Indian J Pediatr 2004;71(11):1015-9.
26. Zlotkin SH, Schauer C, Christofides A, Sharieff W, Tondeur MC, Hyder SM. Micronutrient sprinkles to control childhood anaemia. PLoS Med 2005;2(1):e1.

27. Schauer C, Zlotkin SH. Home fortification with micronutrient sprinkles - A new approach for the prevention and treatment of nutritional anemias. Paediatric Child Health 2003;8(2):87-90.
28. Zlotkin S, Antwi KY, Schauer C, Yeung G. Use of microencapsulated iron(II) fumarate sprinkles to prevent recurrence of anaemia in infants and young children at high risk. Bull World Health Organ 2003;81(2):108-15.
29. Zlotkin S, Arthur P, Antwi KY, Yeung G. Treatment of anemia with microencapsulated ferrous fumarate plus ascorbic acid supplied as sprinkles to complementary (weaning) foods. Am J Clin Nutr 2001;74(6):791-5.
30. Christofides A, Asante KP, Schauer C, Sharieff W, Owusu-Agyei S, Zlotkin S. Multi-micronutrient Sprinkles including a low dose of iron provided as microencapsulated ferrous fumarate improves haematologic indices in anaemic children: a randomized clinical trial. Matern Child Nutr 2006;2(3):169-80.
31. Zlotkin S, Arthur P, Schauer C, Antwi KY, Yeung G, Piekarz A. Home-fortification with iron and zinc sprinkles or iron sprinkles alone successfully treats anemia in infants and young children. J Nutr 2003;133(4):1075-80.
32. Khan E, Hyder SMZ, Tondeur MC, Raza S, Khan NA, Zlotkin S. Home fortification with Sprinkles to reduce childhood anemia: Lessons learned in North West Frontier Province, Pakistan. Pakistan J of Med Res 2006;45(2):35-40.
33. Hirve S, Bhave S, Bavdekar A, Naik S, Pandit A, Schauer C, et al. Low dose 'Sprinkles'-- an innovative approach to treat iron deficiency anemia in infants and young children. Indian Pediatr 2007;44(2):91-100.
34. Menon P, Ruel MT, Loechl CU, Arimond M, Habicht JP, Pelto G, et al. Micronutrient Sprinkles reduce anemia among 9- to 24-mo-old children when delivered through an integrated health and nutrition program in rural Haiti. J Nutr 2007;137(4):1023-30.
35. Zlotkin SH, Schauer C, Owusu Agyei S, Wolfson J, Tondeur MC, Asante KP, et al. Demonstrating zinc and iron bioavailability from intrinsically labeled microencapsulated ferrous fumarate and zinc gluconate Sprinkles in young children. J Nutr 2006;136(4):920-5.
36. Tondeur MC, Schauer CS, Christofides AL, Asante KP, Newton S, Serfass RE, et al. Determination of iron absorption from intrinsically labeled microencapsulated ferrous fumarate (sprinkles) in infants with different iron and hematologic status by using a dual-stable-isotope method. Am J Clin Nutr 2004;80(5):1436-44.
37. Adu-Afarwuah S, Lartey A, Brown KH, Zlotkin S, Briend A, Dewey KG. Randomized comparison of 3 types of micronutrient supplements for home fortification of complementary foods in Ghana: effects on growth and motor development. Am J Clin Nutr 2007;86(2):412-20.
38. Adu-Afarwuah S, Lartey A, Brown KH, Zlotkin S, Briend A, Dewey KG. Home fortification of complementary foods with micronutrient supplements is well accepted and has positive effects on infant iron status in Ghana. Am J Clin Nutr 2008;87(4):929-38.
39. Hyder Z, Zlotkin S. Effect of daily versus once weekly home fortification with sprinkles on haematological and iron status among young children in rural Bangladesh. Food and Nutrition Bulletin 2007;28(2):156-164.
40. Sharieff W, Yin SA, Wu M, Yang Q, Schauer C, Tomlinson G, et al. Short-term daily or weekly administration of micronutrient Sprinkles has high compliance and does not cause iron overload in Chinese schoolchildren: a cluster-randomised trial. Public Health Nutr 2006;9(3):336-44.
41. Sharieff W, Dofonsou J, Zlotkin S. Is cooking food in iron pots an appropriate solution for the control of anaemia in developing countries? A randomised clinical trial in Benin. Public Health Nutr 2008;11(9):971-7.
42. Sharieff W, Bhutta Z, Schauer C, Tomlinson G, Zlotkin S. Micronutrients (including zinc) reduce diarrhoea in children: the Pakistan Sprinkles Diarrhoea Study. Arch Dis Child 2006;91(7):573-9.
43. Zlotkin S. Control of anemia: the time to act is now. Indian Pediatr 2007;44(2):84-6.
44. Sharieff W, Horton SE, Zlotkin S. Economic gains of a home fortification program: evaluation of "Sprinkles" from the provider's perspective. Can J Public Health 2006;97(1):20-3.

45. Christofides A, Schauer C, Zlotkin S. Iron deficiency anemia among children: Addressing a global public health problem within a Canadian context. Paediatric Child Health 2005;10(10):597-601.
46. Christofides A, Schauer C, Zlotkin SH. Iron deficiency and anemia prevalence and associated etiologic risk factors in First Nations and Inuit communities in Northern Ontario and Nunavut. Can J Public Health 2005;96(4):304-7.
47. Christofides A, Schauer C, Sharieff W, Zlotkin SH. Acceptability of micronutrient sprinkles: a new food-based approach for delivering iron to First Nations and Inuit children in Northern Canada. Chronic Dis Can 2005;26(4):114-20.
48. De Pee S, Moench-Pfanner R, Martini E, Zlotkin S, Darton-Hill I, Bloem MW. Home-fortification in emergency response and transition programming: Experiences in Aceh and Nias, Indonesia. Food and Nutrition Bulletin 2007;28(2):189-197.
49. Chandramohan D, Hodgson A, Coleman P, Baiden R, Asante K, Awine E, et al. An evaluation of the immunogenicity and safety of a new trivalent meningococcal polysaccharide vaccine. Vaccine 2007;25 Suppl 1:A83-91.
50. Edmond KM, Kirkwood BR, Amenga-Etego S, Owusu-Agyei S, Hurt LS. Effect of early infant feeding practices on infection-specific neonatal mortality: an investigation of the causal links with observational data from rural Ghana. Am J Clin Nutr 2007;86(4):1126-31.
51. Owusu-Agyei S, Awini E, Anto F, Mensah-Afful T, Adjuik M, Hodgson A, et al. Assessing malaria control in the Kassena-Nankana district of northern Ghana through repeated surveys using the RBM tools. Malar J 2007;6:103.
52. Owusu-Agyei S, Asante KP, Owusu R, Adjuik M, Amenga-Etego S, Dosoo DK, et al. An open label, randomised trial of artesunate+amodiaquine, artesunate+chlorproguanil-dapsone and artemether-lumefantrine for the treatment of uncomplicated malaria. PLoS ONE 2008;3(6):e2530.
53. WHO. World Malaria Report 2008. Geneva, Switzerland; 2008.
54. Chandramohan D, Owusu-Agyei S, Carneiro I, Awine T, Amponsa-Achiano K, Mensah N, et al. Cluster randomised trial of intermittent preventive treatment for malaria in infants in area of high, seasonal transmission in Ghana. Bmj 2005;331(7519):727-33.
55. Verhoef H, West CE, Ndeto P, Burema J, Beguin Y, Kok FJ. Serum transferrin receptor concentration indicates increased erythropoiesis in Kenyan children with asymptomatic malaria. Am J Clin Nutr 2001;74(6):767-75.
56. Labbe RF, Finch CA, Smith NJ, Doan RN, Sood SK, Madan N. Erythrocyte protoporphyrin/heme ratio in the assessment of iron status. Clin Chem 1979;25(1):87-92.
57. McLaren GD, Carpenter JT, Jr., nino HV. Erythrocyte protoporphyrin in the detection of iron deficiency. Clin Chem 1975;21(8):1121-7.
58. Jensen BM, Sando SH, Grandjean P, Wiggers P, Dalhoj J. Screening with zinc protoporphyrin for iron deficiency in non-anemic female blood donors. Clin Chem 1990;36(6):846-8.
59. Labbe RF, Rettmer RL, Shah AG, Turnlund JR. Zinc protoporphyrin. Past, present, and future. Ann N Y Acad Sci 1987;514:7-14.
60. Siegel RM, LaGrone DH. The use of zinc protoporphyrin in screening young children for iron deficiency. Clin Pediatr (Phila) 1994;33(8):473-9.
61. Schifman RB. Red cell protoporphyrin: an alternatie to serum ferritin for assessing iron depletion [Abstract]. Clin Chem 1982;28:1628.
62. Cavill I, Jacobs A, Worwood M. Diagnostic methods for iron status. Ann Clin Biochem 1986;23 (Pt 2):168-71.
63. Asobayire FS, Adou P, Davidsson L, Cook JD, Hurrell RF. Prevalence of iron deficiency with and without concurrent anemia in population groups with high prevalences of malaria and other infections: a study in Cote d'Ivoire. Am J Clin Nutr 2001;74(6):776-82.
64. Stoltzfus RJ, Chwaya HM, Montresor A, Albonico M, Savioli L, Tielsch JM. Malaria, hookworms and recent fever are related to anemia and iron status indicators in 0- to 5-y old Zanzibari children and these relationships change with age. J Nutr 2000;130(7):1724-33.

65. Stoltzfus RJ, Chwaya HM, Albonico M, Schulze KJ, Savioli L, Tielsch JM. Serum ferritin, erythrocyte protoporphyrin and hemoglobin are valid indicators of iron status of school children in a malaria-holoendemic population. J Nutr 1997;127(2):293-8.
66. Rettmer RL, Carlson TH, Origenes ML, Jack RM, Labb RF. Zinc protoporphyrin/heme ratio for diagnosis of preanemic iron deficiency. Pediatrics 1999;104(3):e37.
67. Dimitriou H, Stiakaki E, Markaki EA, Bolonaki I, Giannakopoulou C, Kalmanti M. Soluble transferrin receptor levels and soluble transferrin receptor/log ferritin index in the evaluation of erythropoietic status in childhood infections and malignancy. Acta Paediatr 2000;89(10):1169-73.
68. Ferguson BJ, Skikne BS, Simpson KM, Baynes RD, Cook JD. Serum transferrin receptor distinguishes the anemia of chronic disease from iron deficiency anemia. J Lab Clin Med 1992;119(4):385-90.
69. Olivares M, Walter T, Cook JD, Llaguno S. Effect of acute infection on measurement of iron status: usefulness of the serum transferrin receptor. Int J Pediatr Hematol Oncol 1995;2:31-33.
70. Menendez C, Quinto LL, Kahigwa E, Alvarez L, Fernandez R, Gimenez N, et al. Effect of malaria on soluble transferrin receptor levels in Tanzanian infants. Am J Trop Med Hyg 2001;65(2):138-42.
71. Beguin Y. Soluble transferrin receptor for the evaluation of erythropoiesis and iron status. Clin Chim Acta 2003;329(1-2):9-22.
72. Singhal A, Cook JD, Skikne BS, Thomas P, Serjeant B, Serjeant G. The clinical significance of serum transferrin receptor levels in sickle cell disease. Br J Haematol 1993;84(2):301-4.
73. Musto P, Lombardi G, Centra M, Modoni S, Carotenuto M, Di Giorgio G. Soluble transferrin receptor in beta-thalassaemia. Lancet 1993;342(8878):1058.
74. Rees DC, Williams TN, Maitland K, Clegg JB, Weatherall DJ. Alpha thalassaemia is associated with increased soluble transferrin receptor levels. Br J Haematol 1998;103(2):365-9.
75. Hastka J, Lasserre JJ, Schwarzbeck A, Strauch M, Hehlmann R. Washing erythrocytes to remove interferents in measurements of zinc protoporphyrin by front-face hematofluorometry. Clin Chem 1992;38(11):2184-9.
76. Junca J, Oriol A. Serum transferrin receptor. Am J Clin Pathol 2001;116(3):446; author reply 447-9.
77. Ritchie B, McNeil Y, Brewster DR. Soluble transferrin receptor in Aboriginal children with a high prevalence of iron deficiency and infection. Trop Med Int Health 2004;9(1):96-105.
78. Cook JD, Baynes RD, Skikne BS. Iron deficiency and the measurement of iron status. Nutr Res Rev 1992;5:189-202.
79. Zimmermann MB, Molinari L, Staubli-Asobayire F, Hess SY, Chaouki N, Adou P, et al. Serum transferrin receptor and zinc protoporphyrin as indicators of iron status in African children. Am J Clin Nutr 2005;81(3):615-23.
80. van den Broek N. Anaemia in pregnancy in developing countries. Br J Obstet Gynaecol 1998;105(4):385-90.
81. Pepys MB. C-reactive protein fifty years on. Lancet 1981;1(8221):653-7.
82. Skikne BS, Flowers CH, Cook JD. Serum transferrin receptor: a quantitative measure of tissue iron deficiency. Blood 1990;75:1870-1876.
83. Olivares M, Walter T, Cook JD, Hertrampf E, Pizarro F. Usefulness of serum transferrin receptor and serum ferritin in diagnosis of iron deficiency in infancy. Am J Clin Nutr 2000;72(5):1191-5.

8. PROTECTION OF HUMAN SUBJECTS

8.1 Risks to Human Subjects

8.1a Human Subjects Involvement and Characteristics

- Proposed involvement of human subjects
    i. Studies using animals are not appropriate to answer the research questions posed in this proposal, namely to determine the impact of the provision of iron on the susceptibility to clinical malaria among infants and young children (6-24 months of age)

living in a high malaria burden area, using a powdered vitamin and mineral 'sprinkle' added to complementary foods.

- Characteristics of the subject population – number, age range, health status
    i. Number: Approximately 930 in the intervention group and 930 in the control group
    ii. Age: 6 – 24 months
    iii. Health Status – generally healthy
- Inclusion/exclusion criteria for any subpopulation
    i. Children to be included must meet the criteria described above. They must be free of clinical signs of infection (including malaria) at the time of enrolment. They must have plans to remain in the study area for the duration of the intervention. Informed consent must be freely given by a parent of the children enrolled for the study. Both boys and girls will be recruited without bias. Girl children will specifically be encouraged to participate, as appropriate.
    ii. Children will be excluded if they fail to meet the inclusion criteria shown above.
- Rationale for involvement of special classes of subjects (e.g. children)
    i. The highest malaria burden is in children in the first 3-4 years of life and the highest iron deficiency anemia burden is in the age range 6 – 24 months of age. Thus, we choose to include children who are at highest risk of anemia and malaria.
- List any collaborating sites where human subjects research will be performed, describe role of those sites and collaborating investigators in performing proposed research
    i. The research field work will be conducted in northern Ghana, within the catchment area of the Kintampo Health Research Centre (KHRC). KHRC is one of three Ministry of Health Research Centres in Ghana.
    ii. Research Ethics Board approval will be from two sources: the University of Toronto, the home institution of the PI and the Ghana Ministry of Health.
    iii. The University of Toronto, Hospital for Sick Children PI will provide oversight of the project, while the co-investigator at KHRC will supervise all aspects of the field research.

8.1b Sources of Materials

- Describe research material obtained from individuals in form of specimens, records, or data
    i. Blood samples will be collected from study subjects. These will be in the form of capillary samples and/or venous blood samples. Individuals collecting the samples will be specifically trained in pediatric phlebotomy techniques. Results of analysis of blood samples will be collated and recorded to be used for statistical analysis.
- Describe any data to be collected from human subjects
    i. Individual subject records will be collected. This information includes general demographic data (age and gender) and information specifically related to diet, health and use of health care facilities.
- Indicate who will have access to individually identifiable private information
    i. In Ghana, field workers, field supervisors and the study co-investigator will have access to individually identifiable private information.
    ii. Statisticians, the research coordinator and the PI in Canada will not have access to individually identifiable private information.
- Provide information about how specimens, records, or data are collected and whether will be collected specifically for proposed project
    i. Only data to be included in the statistical analysis to meet primary and secondary objectives will be collected.
    ii. Blood samples are collected as described above.
    iii. Individual data will be collected from the parents or guardians of the study subjects.
    iv. No previous records on study subjects will be collected with the exception of birth weight, immunization status and previous hospitalizations.

8.1c Potential Risks

- Describe potential risks to subjects (physical, psychological, financial, legal, or other), and assess likelihood and seriousness to subjects
    - *i.* Subjects in the intervention group receiving the powdered mineral and vitamin 'sprinkle' *with iron* may be at risk of higher rates of clinical malaria or more severe attacks of malaria or other infections *if they are not anemic* at baseline.
    - *ii.* Subjects in the control group receiving the powdered mineral and vitamin 'sprinkle' *without iron* may be at risk of higher rates of clinical malaria or more severe attacks of malaria or other infections *if they are anemic at baseline.*
    - iii. There are no other psychological, financial, legal or other risks.
- Where appropriate, describe alternative treatments and procedures (including risks and benefits of each)
    - i. All subjects (in both groups) will be provided with treated bed-nets and instructions for their appropriate use. If used, they may decrease the rates of mosquito bites and thus malaria.

8.2 Adequacy of Protection Against Risks

8.2a Recruitment and Informed Consent

- Describe plans for recruitment and process for obtaining informed consent (parental permission and child assent)
    - i. Children between the ages of 6 – 24 months are too young to provide assent.
    - ii. Parents or guardians will be informed of the objectives of the study and the protocol in a setting and language appropriate to rural Ghana. Members of the field research team have worked in the study communities (or neighboring communities) for many years, thus have experience in conducting research in this population.
    - iii. The parent or guardian of the recruited child will be given to opportunity to sign the consent form, or not, in a totally non-coercive manner. Non-participation will have no effect on the provision of care to the child.
    - iv. Permission to recruit in villages in the KHRC catchment area will be obtained from the village assemblies and village elders prior to the start of the recruitment phase.
- Describe circumstances under which consent will be sought and obtained, who will seek it, nature of info provided to subjects, method of documenting consent (justification for waiver if used)
    - i. After permission is granted to recruit from a village (see previous paragraph), individual households will be visited and parents interviewed with regard to inclusion criteria. The study will take place in a very rural environment where telephone and other communication tools are not common. Thus recruitment will be face-to-face.
    - ii. Recruitment will be done by field researchers employed by the Kintampo Health Research Centre. All are trained in health research techniques and many already have experience in recruitment for research projects involving children. Field workers will be supervised by 'field supervisors' who have experience in field research including proper methods of recruitment.
    - iii. Parents of subjects will be provided with oral as well as written material explaining the nature of trial, including procedures, risks and benefits.
    - iv. Consent will be documented through the signing of the name of a parent, or if a signature is not possible, a thumb print will be used.

8.2b Protections Against Risk

- Describe planned procedures for protecting against or minimizing potential risks (e.g. to privacy or confidentiality of data) and assess likely effectiveness
    - i. Data will be kept confidential by ensuring that named documents are locked in cabinets, or if electronically stored, it will be password protected.

   ii. All data will be identified with a numeric-alphabetic code, linked to a master list. It is this master list that will be stored as described above.
   iii. These procedures have been successfully used by the research team in past collaborate research projects without breaches of confidentiality or privacy (to the best of our knowledge).
- Additional protections for children (OHRP subpart D Guidance)
   i. Additional protections for children will be implemented in compliance with NIH policy and as outlined in the applicable sections of 45 CFR Part 46 Subpart D, as well as that deemed necessary by the applicant's Institutional Review Board.
- Plans for ensuring necessary medical or professional intervention in event of adverse effect to subjects
   i. Kintampo Health Research Centre (KHRC) has a well-established clinical facility and referral system to manage all cases (solicited and unsolicited) of adverse reactions. The Kintampo Municipal Hospital which is right in the middle of the district and within 25 kilometers at most from each household has all of the emergency/resuscitation equipment and items to support the clinical trials that KHRC has been embarking on since 2002. This infrastructure and human capacity was set up during early phases of malaria drugs and vaccine trials that KHRC has been involved in. For any complicated cases of malaria or anemia beyond the capacity of the Kintampo Municipal Hospital to handle, an ambulance service is available to facilitate referral to the Komfo-Anokye Teaching Hospital, the second largest Teaching Hospital in Ghana (two hours away). This referral system has been successfully used over the years and will be available for all children participating in this trial.

8.3 Potential Benefits of the Proposed Research to Human Subjects and Others

- Discuss potential benefits of research to participants and others
   i. Subjects in the intervention group may benefit from the intervention by having fewer bouts of anemia or less severe attacks of malaria or other infections *if they are anemic* at baseline.
   ii. Subject in the control group may benefit from the intervention by having fewer bouts of anemia or less severe attacks of malaria or other infections *if they are non-anemic* at baseline.
   iii. The results of this research may ultimately benefit the children of Ghana (and other West African countries) by informing policy on the appropriate use of iron in children in high malaria burden areas at risk of iron deficiency anemia.
- Discuss why risks to subjects reasonable in relation to anticipated benefits
   i. There is relative equipoise for subjects in the control and intervention groups for many reasons. Subjects with iron deficiency anemia may benefit from the powdered iron supplement and subjects without iron deficiency may benefit from being in the control group. Risks are similarly equal among the two groups.
   ii. All subjects will receive treated bed nets which will mitigate the risk of contracting malaria.

8.4 Importance of the Knowledge to be Gained

- Discuss importance of knowledge to be gained
   i. Currently there is great confusion regarding the safety of providing iron to children at high risk of iron deficiency anemia in a high burden malaria area. If a child with severe iron deficiency anemia remains untreated, death may ensue. With moderate or even mild iron deficiency anemia, there is documentation of adverse developmental

consequences including delayed motor and cognitive development that may not be reversible.

ii. The decision to withhold iron supplements (UNICEF/WHO guidance) was based primarily on a single study from Zanzibar in a highly malaria endemic region, where bed nets were neither provided nor widely used. In that study it was the children without anemia who fared the worst.

iii. The knowledge to be gained from this study may help inform public policy on this important issue.

- Discuss why risks to subjects are reasonable in relation to importance of knowledge that may be expected to result
    i. In most developing countries it is not possible to screen children for iron deficiency anemia. The currently available tools for screening are insensitive and often more costly than the intervention. Thus, it is recommended that when the prevalence of anemia is greater than 40%, blanket fortification or supplementation is warranted.
    ii. As previously noted, it is the children who do NOT have iron deficiency anemia that seem to be at higher risk of adverse outcomes if they are given iron and concomitantly have malaria. But this observation is primarily from a single study.
    iii. In the current study, because of the design, there is a risk of giving iron or not giving iron depending on the individual circumstances of the child. However, the form of iron (microencapsulated iron) and the delivery system (as a powder and added to food), may protect all children from adverse effects of iron, even if they have malaria.
    iv. The information that hopefully will be obtained from the results of this study has the potential to be very important for forming public policy regarding the use of iron in a high malaria burden area.

8.5 Data and Safety Monitoring Plan

- General description of a monitoring plan that will establish as the overall framework for data and safety monitoring. Describe entity that will be responsible for monitoring and the process by which Adverse Events will be reported to the Institutional Review Board, the funding I/C, the NIH Office of Biotechnology Activities (OBA), and the Food and Drug Administration (FDA) in accordance with Investigational New Drug (IND) or Investigational Device Exemption (IDE) regulations.
    i. Field workers will describe any possible adverse events to the field supervisors. At weekly meetings with the study site coordinator, potential adverse events will be reviewed. A written summary of this weekly meeting will be shared with the co-investigator who will decide if the information should be shared with the PI. Any adverse events reported to the PI will be reported to the IRBs in Ghana and Canada.
- Options for monitoring trials include, but not limited to: PD/PI (required); Institutional Review Board (required); Independent individual/safety officer; Designated medical monitor; Internal Committee or Board with explicit guidelines; Data and Safety Monitoring Board (required by NIH for multi-site clinical trials involving interventions that entail potential risk to participants, and generally for Phase III clinical trials)
    i. A detailed Data and Safety Monitoring Plan will be submitted to IRBs in both Toronto and Ghana and (subsequently) the funding IC for approval prior to the accrual of human subjects.

8.6 ClinicalTrials.gov Requirements

- NIH encourages registration of ALL trials in ClinicalTrials.gov whether required under the law or not.
    i This trial will be registered at ClinicalTrials.gov

9. INCLUSION OF WOMEN AND MINORITIES

This project is specifically directed to children in the age range 6 – 24 months, thus, women will not be recruited to be included as subjects. In most cases, however, the mothers of the children will contacted as the surrogate to provide consent for their children to be included in the study. All ethnic, racial and minority groups will be included in the recruitment and the study.

10. TARGETED/PLANNED ENROLMENT TABLE

Not applicable

11. INCLUSION OF CHILDREN

This project is specifically directed to children in the age range 6 – 24 months. This age range was chosen because it is considered to be both the most vulnerable period of life for the infant and young child, and also a 'window of opportunity'. It is vulnerable because growth is faster during this period than at any other time in the life of the child, thus the need for nutrients is higher during this time. If individual nutrients are missing from the child's diet during this time period, the consequences can be long-lasting. For example, there is documentation that young children diagnosed with iron deficiency anemia during the first year of life were at risk of retarded development in certain key areas, including some emotional development and educational attainment. This age range is also specifically pertinent to the questions posed in the RFA, since it refers to the recent study conducted in Pemba, which included only infants and young children.

Both investigators (Owusu and Zlotkin) have experience in performing research in infants and young children. Zlotkin specifically is a pediatrician. His clinical and research focus is on children. His laboratory is at the Hospital for Sick Children in Toronto, and the IRB is located at the Hospital for Sick Children. The Hospital for Sick Children is the largest children's hospital in Canada, and possibly in North America, and has one of the largest Research Institutes associated with it. The IRB at the Hospital and Research Institute is thus extremely well-suited to evaluate the ethical aspects of research involving children. Field researchers at the Kintampo Health Research Centre (KHRC) have experience working with children and their parents, and phlebotomists at KHRC are experienced in taking blood samples from children. Further, there are sufficient numbers of children in the catchment area of KHRC to meet the sampling needs of the study. The fertility rate in Ghana is 3.78 children born/women (2008 estimate) (https://www.cia.gov/library/publications/the-world-factbook/geos/gh.html).

12. VERTEBRATE ANIMALS

Not applicable

13. SELECT AGENT RESEARCH

Not applicable

14. MULTIPLE PD/PI LEADERSHIP PLAN

This project will be a joint effort between the University of Toronto, the Research Institute at Hospital for Sick Children, and the Ghanaian Ministry of Health, through the Kintampo Health Research Centre (KHRC). The PI from the University of Toronto, Department of Paediatrics, Nutritional Sciences and Public Health Sciences has extensive experience in research involving children, specifically research on iron and anemia in developing

countries, including Ghana. The co-investigator is the Director of the KHRC. He has extensive research experience dealing with malaria, including iron and malaria, in Ghana. Drs. Zlotkin and Owusu have worked successfully together in the past, thus one of the advantages of this collaboration is the trust between the two institutions and individuals running the study.

As has been the case with past studies involving the two institutions, there will be a clear *a priori* communications plan. For the weeks preceding the start of the study, there will be an on-site presence of the PI and/or his delegate. There will be weekly conference calls for the first 4-6 weeks of the study, and bi-weekly calls thereafter. There will be routine e-mail communication between the study co-coordinator in Toronto and her equivalent in Kintampo. All important decisions about the protocol and procedures will have been made before the protocol is submitted to the NIH. Ongoing major decisions will be made jointly by the PI and co-investigator, as has been the case in past studies. Any potential differences in opinion will be worked out through discussion and compromise. Publications will be jointly authored; with Dr. Owusu as the primary author on those publications pertaining to malaria, and Dr. Zlotkin as the primary author on those pertaining to anemia.

IRB reviews will be completed in both Ghana and Canada. In Canada, Dr. Zlotkin will be responsible for shepherding the review, while Dr. Owusu will be responsible in Ghana. The budget allocation (Ghana vs. Canada) is clearly shown in the budget component of this application.

15. CONSORTIUM/CONTRACTUAL ARRANGEMENTS

An agreement including programmatic, fiscal and administrative arrangements will be developed and signed by both organizations (the Kintampo Health Research Centre and the Research Institute at the Hospital for Sick Children). Similar agreements have been used in past collaborative projects between the two organizations.

16. LETTERS OF SUPPORT

See the attached letter of support from the consortium.

17. RESOURCE/DATA SHARING PLAN

Findings from the proposed research will be shared according to the applicable NIH policy for foreign institutions.
